# A ubiquitous method for predicting underground petroleum deposits based on satellite data

**DOI:** 10.1038/s41598-023-32054-0

**Published:** 2023-04-24

**Authors:** Sarfaraz Newaz, Md Toki Tahmid, Nadia Al-Aboody, A. B. M. Alim Al Islam

**Affiliations:** 1 Next-generation Computing (NeC) Research Group, Department of Computer Science and Engineering, Bangladesh University of Engineering and Technology, Dhaka, Bangladesh; 2 Department of Computer System, Amarah Technical Institute, Southern Technical University, Basrah, Iraq

**Keywords:** Energy harvesting, Energy storage, Natural gas, Petrol, Mineralogy, Petrology

## Abstract

The method of finding new petroleum deposits beneath the earth’s surface is always challenging for having low accuracy while simultaneously being highly expensive. As a remedy, this paper presents a novel way to predict the locations of petroleum deposits. Here, we focus on a region of the Middle East, Iraq to be specific, and conduct a detailed study on predicting locations of petroleum deposits there based on our proposed method. To do so, we develop a new method of predicting the location of a new petroleum deposit based on publicly available data sensed by an open satellite named Gravity Recovery and Climate Experiment (GRACE). Using GRACE data, we calculate the gravity gradient tensor of the earth over the region of Iraq and its surroundings. We use this calculated data to predict the locations of prospective petroleum deposits over the region of Iraq. In the process of our study for making the predictions, we leverage machine learning, graph-based analysis, and our newly-proposed OR-nAND method altogether. Our incremental improvement in the proposed methodologies enables us to predict 25 out of 26 existing petroleum deposits within the area under our study. Additionally, our method shows some prospective petroleum deposits that need to be explored physically in the future. It is worth mentioning that, as our study presents a generalized approach (demonstrated through investigating multiple datasets), we can apply it anywhere in the world beyond the area focused on in this study as an experimental case.

## Introduction

The world runs on energy, without which the world could be near obsolete. With the industrial revolutions and inventions of energy-driven machinery, we now rely more on energy to run things than ever. The energy is produced mostly from petroleum such as fossil oils. However, human beings are yet to produce petroleum even with the latest and the most promising research to date. This forces us to search for petroleum beneath the earth’s surface, which gets developed through million-years-long geological formations. After finding and extracting petroleum from underground, we refine it into various types of fuels for real usage.

Different types of technologies are currently used for finding petroleum deposits^1–3^. Most of them are highly expensive and less accurate. Besides, the existing technologies generally need drilling to check if there is any petroleum deposit or not^3^. Therefore, finding new petroleum deposits generally demands a huge budget^4^. In this context, prior computing-based prediction to facilitate finding a new petroleum deposit incurring a low cost and resulting in high accuracy has become very important in today’s world. For making such computing-based predictions, data pertinent to existing petroleum deposits are required. A large number of existing petroleum deposits are located in the Middle East, where a prominent representative country is Iraq. As petroleum deposits are a good property for the geology of Iraq^5^, therefore, computing-based predictions of underground petroleum deposits based on data on the existing deposits in Iraq and its surrounding places are worth investigating, which we focus on in this study.

To explore similar contexts, numerous research studies have been performed for detecting petroleum deposits all over the world. These existing research studies leverage physical interventions. For example, in a conventional approach, a petroleum deposit is first guessed from accumulated experience, and then exploratory well drilling takes place^6^. The guess of having a petroleum deposit is based on previous experience with geological structures such as anticlines, which often proves to be inaccurate after performing the drilling^6^. In reality, the task of drilling is highly expensive even though it is onshore^4^. To better portray how costly the drilling is, we present Table 1 showing estimates of the costs of drilling a well more than a decade back covering both onshore and offshore.

**Table 1:** Drilling costs in different settings based on data presented in^4^.

Rig Location Type	2010 Rig Rate ($/day)	Depth (ft)	Drilling Days	Well Cost (Million $)
Onshore	<100K	20,000	70–80	7–8
Onshore	<100K	26,000	110	11
Onshore	<100K	32,000	150	15
Offshore	500–800K	20,000	70–80	35–64
Offshore	500–800K	26,000	110	55–88
Offshore	500–800K	32,000	150	75–120

Considering the cost of drilling, researchers have got interested in using remote sensing and geographical information system (GIS) to find the potential areas of petroleum deposits. If an area is found to be a potential one in this way, seismic technology becomes worth using for further exploration. Following this approach, the studies in^7,8^ used locally-sensed gravity information to find a probable area of a new petroleum deposit. Apart from searching petroleum deposits in this way, remote sensing techniques are also used in different other environmental studies owing to the promise the remote sensing techniques exhibit.

Considering these aspects, in this paper, we further leverage the notion of remote sensing by utilizing satellite data. Here, we use the gravity gradient tensor calculated from the data sensed by the GRACE satellite to predict a new potential petroleum deposit. Since the data sensed by the GRACE satellite appear to be open-source and the data are available for the whole world, our study based on GRACE satellite data presents a generalized computational method for predicting petroleum deposits.

In our study, we first take the gravity data (sensed by the GRACE satellite) of our area of focus and its surroundings. We consider the surroundings as the training regions and take the area under focus as our testing region. In parallel, we take the locations of the already-discovered petroleum deposits over the training and testing regions from the Peace Research Institute of Oslo (PRIO)^9^. Then, we combine the gravity data of our training and testing regions with the locations of already-discovered petroleum deposits to build our training and testing datasets. In the datasets, the total number of locations having no petroleum deposits is much higher than that having petroleum deposits, which results in imbalanced datasets having only a handful of positive cases. This is also the reason why we could not use deep learning in our study. To overcome the problem of having imbalanced datasets, we oversample our training datasets using standard oversampling techniques to make them balanced. Subsequently, we apply 28 different machine-learning methods to our training datasets and find out the best-performing machine-learning methods among them. Then, we apply the models found from the best-performing machine learning methods on our testing datasets and analyze their performances. We find that there exists no machine learning method among our explored ones whose output model can give an expected level of performance on our testing data. To this end, we understand that none of the existing machine learning models can solely appear to be promising in detecting underground petroleum deposits.

Next, to go further, we propose a new method realizing a combination of OR-ing and then n-times ANDing. The method leverages the notion of n-voting over the best-performing models, and thus, improves the performance of predicting underground petroleum deposits. We name this method as OR-nAND method. Besides, we perform graph-based analysis to minimize the number of considered best-performing models on which our proposed OR-nAND method is applied. Additionally, we present pictorial views of our findings on prospective undiscovered petroleum deposits in Iraq through heatmaps. Finally, we explore the whole method over another dataset from Harvard ArcGIS WorldMap^10^ to demonstrate the generalizability of our proposed method. While experimenting with the dataset from Harvard ArcGIS WorldMap, we find similar outcomes as already obtained for the dataset from PRIO.

In the process of this study, we make the following set of contributions in this paper.

- We take gravity information from the GRACE satellite and existing petroleum deposits over Iraq as well as its surrounding areas. We combine the information to generate 80 different training datasets and four testing datasets.
- We conduct oversampling methods over our training datasets to make them balanced. We apply 28 different machine-learning methods over each of the balanced datasets and find out the best-performing machine-learning methods.
- We apply the models found from the best-performing methods over the testing datasets and find low accuracy there. To ameliorate the level of performance, we further propose a new method named OR-nAND by utilizing the notions of n-voting and graph-based analysis.
- Further, to demonstrate the generalizability of our proposed method, we explore the whole method over another dataset from Harvard ArcGIS WorldMap and demonstrate similar findings.
- Finally, we apply our improved models over Iraq to predict potential petroleum deposits that are yet to be explored. We present our findings using heatmaps to make them easily understandable.

The rest of the paper is organized as follows: “Related Work and Gap in the Literature” section presents related research studies in this field. In “Methodology of Our Study” section, we elaborate the methodology of our study. We describe our experimental setup in “Experimental Setup” section. “Results and Findings” section presents our experimental results. We further discuss our findings in “Discussion” section. Finally, in “Future Work” and “Conclusion” sections, we point out scopes of future research and then conclude our paper.

## Related Work and Gap in the Literature

Our study presented in this paper subsumes three different perspectives: petroleum deposits and their predictions, the use of satellite data in studying the properties of the earth, and satellite-based study over and around the region of Iraq. Therefore, we present our related research studies from these three perspectives below.

### Petroleum Deposits and Their Predictions

Predicting a petroleum deposit is always challenging. A number of research studies have been done focusing on this problem. For example, Aghajani et al. tried to detect high-potential petroleum deposits using normalized full gradients of gravity anomalies^7^. Their area under study was the Tabas basin of Eastern Iran. Similarly, Zeng et al. also tried to detect reservoirs using normalized full gradients of gravity anomalies. They applied their method to the Shengli oil field of East China^8^. Both of these studies share common limitations - they worked with data from a specific source (not an open source) and they worked with localized custom data. For example, Aghajani et al., managed the localized gravitational data from the geophysics department of the National Iranian Oil Company^7,11^. Besides, Zeng et al. collected the gravitational data from the Exploration Company of Shengli Petroleum Administration Bureau^8^. These data sources deal with localized data and are not openly accessible to all. On the other hand, in our study, we use open GRACE satellite data as the source of the gravitational data covering the whole earth. Using this gravitational data, we calculate the gravity gradient tensor in our study.

Besides, various automated and mathematical modeling-based approaches have been proposed over the years for exploring underground oil and gas reservoirs. The study in^12^ proposed a controlled-source electromagnetic data analysis-based method, which explores locating the right depth of reservoirs in oil field areas. Besides, the study in^13^ investigated low-temperature thermochronology-based techniques in the exploration of hydrocarbons. Additionally, the study in^14^ focused on searching for sweet spots to locate the most optimized drilling location in a reservoir. The study in^15^ introduced gradient-boosting decision trees (GBDTs) to automatically determine sweet spots based on well-log data sets. Similarly, the study in^16^ explored a model to locate the optimum position of wells in an underground reservoir. In another study^17^, a well located in the Nias Basin (in the west of Sumatra) is studied using geochemical data. In addition, the study in^18^ performed medium-term forecasting of salinity rates and groundwater levels using statistical and machine learning-based methods. Nonetheless, the study in^19^ explored forecasting of thermal regimes in oil fields by developing a differential equation-based mathematical model to describe the process of formation of thermal conditions in a mine.

### Satellite Data Mining

Several research studies have been performed using satellite data mining. For example, Gido et al. used the GRACE data in the study of existing oil fields in Sudan. They did not attempt to detect or predict a new oil field, rather they studied ground subsidence due to the extraction of groundwater and oil from the existing oil deposits^20^. Nabaz et al. used remotely sensed Landsat satellite imagery, geographic information systems, and the hybrid cellular automata (Markov model) to study the region of Sulaimani Province in Kurdistan, Iraq^21^. Before them, Rahel et al. did a similar work over the Halgurd-Sakran Core Zone of the National Park in the Kurdistan, Iraq^22^ using Landsat-5 and Landsat-8 images in association with the Cellular Automata (Markov chain) model. Besides, using the Landsat-8 and Markov-Cellular Automata, Emran et al. showed the degradation of the world’s largest mangrove forest and predict the forest cover^23^. Additionally, Satellite imagery obtained from Landsat-8 was also used to detect and study oil slicks to get a deep insight into oil pollution in the Arabian Gulf and the Sea of Oman^24^ by Zhao et al. Nonetheless, remote sensing and GIS have been used in different research studies. For example, Naji et al., used remote sensing and GIS for spatial analysis of the chemical soil properties of South Basra, Iraq^25^. Moreover, remote sensing and GIS are also used to study sand and dust storms in the Middle East^26–29^. However, none of these studies on detecting petroleum deposits are performed based on the property of the earth sensed by a satellite.

### Satellite-based Studies over and around Iraq

Behadili et al. investigated Landsat-7 data for the Al-Nasiriya city of Iraq^30^. They looked at thermal bends from satellite images to study the extraction of emitted hydrocarbon. From this emission, they tried to find out unexplored oil and gas fields^30^. Besides, Perry et al. used multispectral satellite imagery over the region of Kurdistan, Iraq to detect hydrocarbon seepage^31^. Additionally, Omar et al. extracted tectonic linaments from Landsat-7 imagery in the Tawke oil field, Kurdistan, Iraq^32^. On the other hand, Allafta et al. performed a GIS-based analysis for flood-prone area mapping along the boundary of Iraq-Iran using satellite images^33^. All of these research studies primarily focused solely on satellite images. Thus, extracting the property of the earth from satellite data for the purpose of detecting petroleum deposits is yet to be investigated in the literature.

## Methodology of Our Study

**Figure 1:**
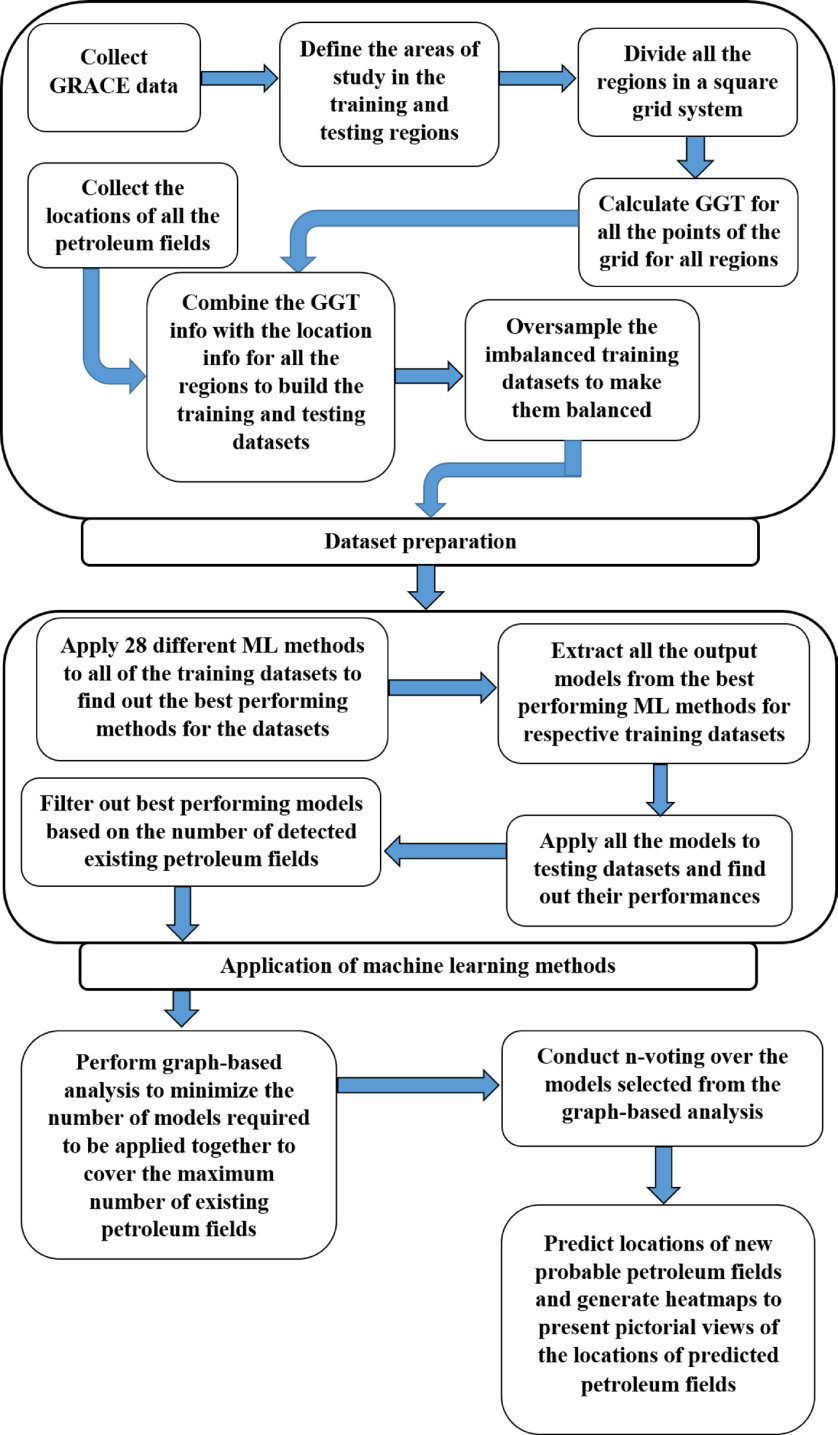
Methodology and different steps performed in the study.

We perform our study following a number of steps. At first, we build our training and testing datasets. Building the datasets subsumes a combination of steps. After building the datasets, we apply a number of machine learning (ML) methods to each of the training datasets. Subsequently, we take the best-performing ML method for each training dataset and apply the best-performing model to the corresponding testing dataset. We find that the best-performing models do not come from any single ML method, and therefore, no single ML method can always exhibit the best performance. All of the best-performing models found in this way can predict some of the existing petroleum fields in the testing datasets. We, therefore, build a matrix based on the performances of the best-performing models over corresponding testing datasets. In the matrix, for each best-performing ML model, we pinpoint the petroleum deposits in the corresponding testing dataset that are predicted by the model and count the total number of petroleum deposits predicted by the model. Then, we sort the matrix based on the total number of predicted petroleum deposits. Afterward, we apply graph-based analysis to find out the minimum number of ML models that should work together to provide a good prediction performance. Next, we apply n-voting over the outcomes of the ML models selected from the outputs of the graph-based analysis and find substantial accuracy in prediction. Here, while performing the graph-based analysis, we take the models that combinedly choose the existing petroleum deposits by being predicted by at least one of the models. This mimics the notion of an OR operation and therefore, we refer to this operation as OR-ing in our case. Then, we apply n-voting for finding the most probable new petroleum deposit through performing n times AND operation over the output of the selected models, Thus, we refer to this operation as nANDing in our case. Note that, when a point is selected as a probable new petroleum deposit by n-voting, the point may be selected by any set of the n models. Accordingly, two different points may be selected by different sets of n models. Here, the models that select the probable points can be completely different. However, the count of the selector model is at least n. In other words, nAND-ing is not fixed for any specified ML models, rather, it is fixed on the count of the ML models giving the prediction. That is why we name our overall proposed method as OR-nAND method. Finally, we convert our findings into pictorial views using heatmaps presenting predicted new petroleum deposits. Figure 1 shows the methodology in a flowchart form. We describe each part of the methodology in detail below.

### Dataset Preparation

One of the basic building blocks of our dataset is the data sensed by GRACE satellites. GRACE satellites provide us with a monthly average of Earth’s spherical harmonic coefficients. Since the earth is like a sphere, we can calculate various properties of the earth, e.g., the gravitational field, magnetic field, etc., using these spherical harmonic coefficients using Legendre polynomials^34^.

The gravitational field of any point on the earth can be computed as follows.

1 $$\begin{aligned} V=\frac{GM}{r} \times \Bigg (\sum _{n=2}^{\infty }\sum _{m=0}^{n}\bigg (\frac{a_e}{r}\bigg )^n \times \left( C_{nm}\times \cos {(m\lambda )}+S_{nm}\times \sin {(m\lambda })\right) \times P_{nm}\times \cos \theta \Bigg ) \end{aligned}$$

here $C_{nm}$ and $S_{nm}$ are the spherical harmonic (SH) coefficients^35^, which describe the mass distribution within the earth. $a_e$ is the equatorial radius. *r*, $\theta$, and $\lambda$ are the radius, colatitude, and longitude respectively. $P_{nm}$ is the associated Legendre function. *GM* is the gravitational constant multiplied by the mass of the earth.

On the other hand, the earth’s continental crust has an average density of 2900 kgm$^{-3}$^36^. Where any of the underground petroleum reservoirs exist, the density of the earth of that place will be less than the other places of surroundings having no reservoirs^37^. Due to the relationship with density, the gravitational field will face some curve in the spatial domain at that place^38^. Since this gravitational change will be extremely small, a more detailed sensitive property called Gravity Gradient Tensor (GGT)^39^ can be used to detect such anomalies in the gravitational field in the spatial domain.

Gravitational force in a gravitational field can be divided into three components in three directions. Each of them can be divided again into three directions, which is the second derivative of the gravitational force in the NED (North-East-Down) frame^35^. As a result, among a total of nine components, six are unique and named Gravity Gradient Tensors (GGT)^40^. Since it is the second derivative of the gravitational force, it is more sensitive in each direction^41^. This property of the earth can be used to detect very small anomalies in gravitational fields in the spatial domain, which can in turn towards detecting some prospective petroleum deposits. Note that, GGT components can be measured only for specific locations on earth, i.e., for any specific latitude and longitude value. To meet this requirement, we need to divide the earth into a grid system.

To further elaborate this study, we first calculate the gravity gradient tensor (GGT), which has six components. Those components are Vxx, Vxy, Vxz, Vyy, Vyz, and Vzz. It is important to keep in mind that GGT components cannot be computed generally^42^. They can be calculated only for specific locations on earth, i.e., for specific values of latitude and longitude. To meet this requirement, we need to divide the area under our study into a grid system. Then, GGT components can be calculated for each point of that grid system.

To calculate the GGT for a specific point on the grid system, we first need the first derivatives of Eq. 1, and then the second derivatives of it. Therefore, based on Eq. 1, the first derivatives of *V* with respect to *r*, $\theta$, and $\lambda$ are calculated first as $V_r(r,\theta ,\lambda )$, $V_{\theta }(r,\theta ,\lambda )$, and $V_{\lambda }(r,\theta ,\lambda )$. Accordingly, the second derivatives of *V* with respect to *r*, $\theta$, and $\lambda$ are calculated then as $V_{rr}(r,\theta ,\lambda )$, $V_{r\theta }(r, \theta , \lambda )$, $V_{r\lambda }(r, \theta , \lambda )$, $V_{\theta \theta }(r, \theta , \lambda )$, $V_{\theta \lambda }(r, \theta , \lambda )$, and $V_{\lambda \lambda }(r, \theta , \lambda )$. And then, the gravitational gradients full tensor (GGT) in the local North-East-Down (NED)^35^ frame can be further derived as follows.

2 $$\begin{aligned}{} & {} V_{xx}(r, \theta , \lambda ) = \frac{1}{r}V_r(r, \theta , \lambda ) + \frac{1}{r^2}V_{\theta \theta }(r, \theta , \lambda ) \end{aligned}$$

3 $$\begin{aligned}{} & {} V_{xy}(r, \theta , \lambda ) = V_{yx}(r, \theta , \lambda ) = \frac{1}{r^2\sin {\theta }}(-\cot {\theta }V_{\lambda }(r, \theta , \lambda ) + V_{\theta \lambda }(r, \theta , \lambda )) \end{aligned}$$

4 $$\begin{aligned}{} & {} V_{xz}(r, \theta , \lambda ) = V_{zx}(r, \theta , \lambda ) = \frac{1}{r}V_{r\theta }(r, \theta , \lambda ) - \frac{1}{r^2}V_{\theta }(r, \theta , \lambda ) \end{aligned}$$

5 $$\begin{aligned}{} & {} V_{yy}(r, \theta , \lambda ) = \frac{1}{r}V_r(r, \theta , \lambda ) + \frac{1}{r^2}\cot {\theta }V_{\theta }(r, \theta , \lambda ) + \frac{1}{r^2\sin ^2{\theta }}V_{\lambda \lambda }(r, \theta , \lambda ) \end{aligned}$$

6 $$\begin{aligned}{} & {} V_{yz}(r, \theta , \lambda ) = V_{zy}(r, \theta , \lambda ) = \frac{1}{r\sin {\theta }}\bigg (V_{r\lambda }(r, \theta , \lambda ) - \frac{1}{r}V_{\lambda }(r, \theta , \lambda )\bigg ) \end{aligned}$$

7 $$\begin{aligned}{} & {} V_{zz}(r, \theta , \lambda ) = V_{rr}(r, \theta , \lambda ) \end{aligned}$$

The equations needed to calculate those GGT components are given in the Supplementary Material in more detail. From Eq. 1, we can find two terms $C_{nm}$ and $S_{nm}$ in the calculations. These are spherical harmonic coefficients of the earth. GRACE satellite provides monthly spherical harmonics of earth, i.e., $C_{nm}$ and $S_{nm}$ data. Using these satellite data and equations, we calculate the GGT components for a specific point on the grid system.

Then, we calculate the GGT for every point on the grid. It is worth mentioning that, calculating GGT components is a time-consuming job. Besides, in our study, we need the value of a GGT component for the same point many times for the shake of our experiment. To overcome the repetition of the time-consuming calculation of GGT on the road to save the total time of our experiment, we first calculate GGT components for all the points of the grid system under our study area and save all of them in storage. Then, we take the values from the storage during experimentation following the notion of dynamic programming way.

In this way, we calculate all six GGT components for our area of focus which covers Iraq and its surroundings. Based on these GGT components, our plan is to learn the properties of the earth from gravitational anomalies in the surroundings of Iraq. We want to use the learned model for detecting new prospective petroleum deposits over the region of Iraq.

It is worth mentioning that, in comparison to the number of non-petroleum locations, the number of already known petroleum deposit locations is substantially lower, Therefore, we cannot use the deep learning method here. Besides, we observed that the datasets appear to be imbalanced datasets. Therefore, before performing any processing, we need to balance the datasets using standard oversampling techniques. After performing the oversampling, we plan to apply several standard machine learning methods and find out which methods perform best for our oversampled training datasets. After getting the trained models from the best-performing methods for the adjacent and surrounding areas of Iraq, we will apply those models over the region of Iraq and try to predict if there is any new prospective petroleum deposit in Iraq.

**Figure 2:**
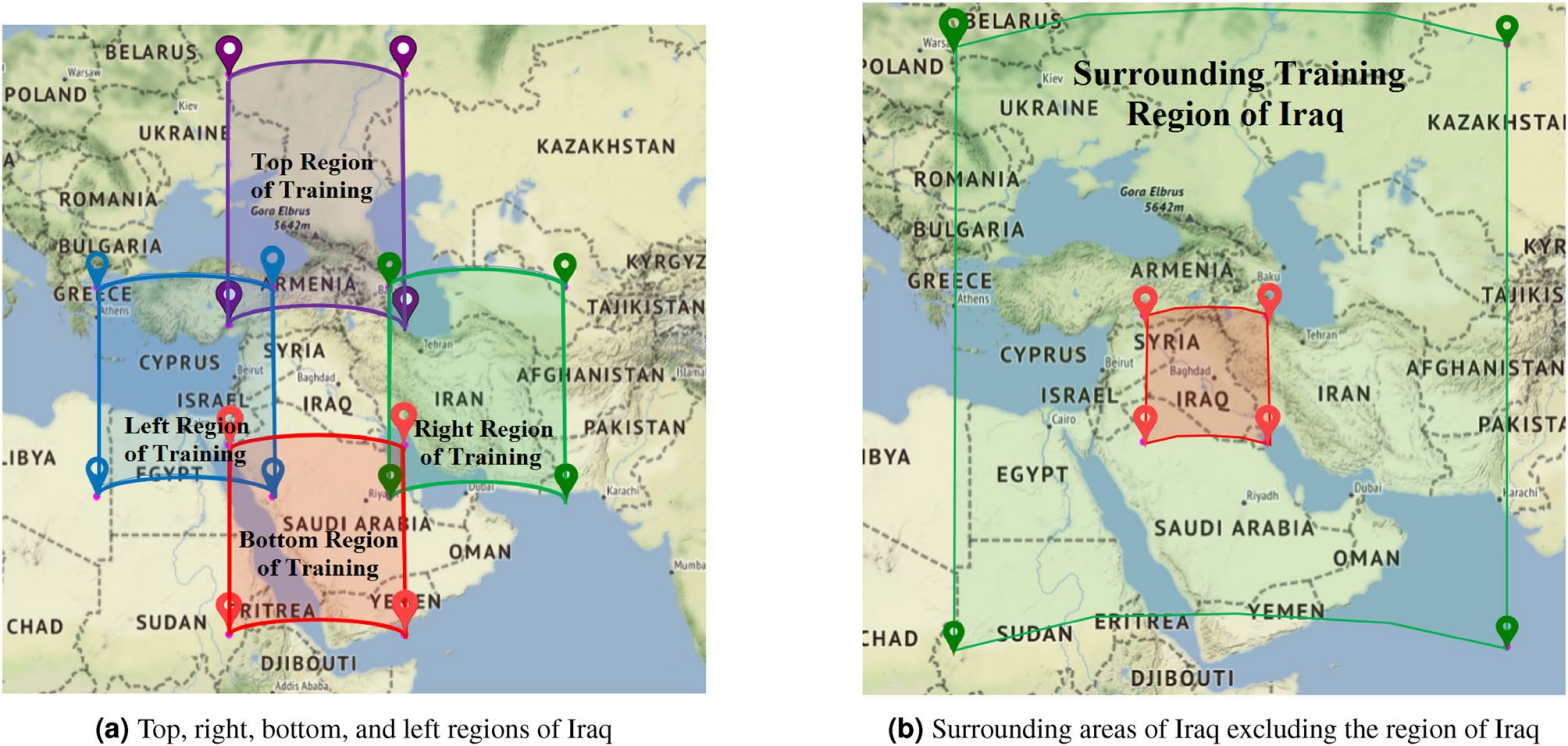
Regions that are considered in preparing training datasets (the figures are created using Python 3.8^43^ and Microsoft Power Point^44^).

To focus on our prediction over the region of Iraq and to make our training and testing datasets disjoint, we take four $15^\circ \times 15^\circ$ regions adjacent to Iraq. We take these four adjacent regions having the same size in terms of degree and being adjacent to the north, east, south, and west of Iraq. Figure 2a presents these four adjacent regions used for training in our experiment. For each of these regions, we take the locations of existing petroleum deposits of respective regions and then use them in building our training datasets.

Additionally, we consider the four adjacent regions as a whole. To do this, we take the west-most point of the training region at a distance of $15^\circ$ west from the west-most point of Iraq. Consequently, we take the east-most point of the training region at a distance of $15^\circ$ east from the east-most point of Iraq. Similarly, we take the northern-most and southern-most points of the training region at a distance of $15^\circ$ north and $15^\circ$ south from the north-most and south-most points of Iraq respectively. We consider this whole surrounding region of Iraq as a training region. Note that, in this case, we carefully exclude the middle part of the region, which is the Iraq region, to make sure that our training and testing datasets remain disjoint. Figure 2b presents the surrounding training region of our experiment. Like the other four adjacent regions shown in Fig. 2a, we also take the locations of petroleum deposits over this surrounding region to build our training datasets.

It is worth mentioning that, for one single region, the number of petroleum deposits is substantially lower than that of available non-petroleum points in that region. This gives us an imbalanced dataset. To overcome this problem, we apply standard oversampling techniques to our primary imbalanced datasets and get balanced training datasets. We use both SMOTE^45^ and ADASYN^46^ as oversampling techniques in our study.

Further, we prepare four testing datasets in exactly the same way as the training datasets get prepared. Here, while preparing the testing datasets, the region and the testing petroleum deposits remain the same. However, as we take data from four different data sources, we end up with four testing datasets. Note that, while applying the models on the prepared testing datasets, we always carefully maintain applying the appropriate ML models on the testing datasets in such a way that the data source for the training and testing datasets remains the same. Otherwise, the result would be invalid. Thus, the main difference in preparing the testing datasets in comparison to preparing the training datasets is that, in the case of the testing datasets, the area under consideration is Iraq itself. Moreover, oversampling is not needed for the testing datasets, as we do not perform any training using these datasets.

### Application of Machine Learning Methods

After preparing the training datasets, we apply different machine learning (ML) methods to each of them with various time durations for training. Time durations are multiple of 15 minutes, starting from 15 minutes up to 1920 minutes. Among all of them, we take the best-performing ML methods for each of the training datasets. The best-performing ML methods vary from one dataset to another. Therefore, we apply all the models extracted from the best-performing ML methods to the respective testing datasets. For each of the models, we find that they can predict different petroleum deposits from our testing datasets.

Note that, none of the best-performing models predict all of the petroleum deposits in our testing datasets. In fact, each one can predict some of the existing petroleum deposits. However, we notice that the petroleum deposits predicted by different resulting models are not unique for each of the resulting models even for the models that predict the same number of petroleum deposits. If the number of predicted petroleum deposits is not the same, the predicted petroleum deposits obviously vary for different resulting models. To analyze which petroleum deposit in a testing set is predicted by which model and which petroleum deposits are predicted most of the time, we prepare a matrix. In the matrix, we take the number of columns as the number of petroleum deposits in the testing region. Besides, we take all the results on predicting existing deposits in the testing datasets by all the models as the rows of the matrix. Then, we row-wise sort the matrix in descending order based on the total number of predicted petroleum deposits by the models. We find that some of the petroleum deposits cannot be predicted by any of the models. Besides, some of the models predict only a few petroleum deposits. Considering all these aspects, we need a threshold on the number of predicted petroleum deposits by a single model based on which we can choose a set of models in the process of making the final decision. To determine the threshold, we build a table, each row of which contains three things-1) The minimum number of petroleum deposits predicted by a single model, 2) Which petroleum deposits can be predicted for how much time by combining all the models that can predict at least the minimum number of existing petroleum deposits, and 3) The total number of predicted petroleum deposits by combining all these models that can predict the minimum number of existing petroleum deposits. From this table, we plot a graph and find the value of saturation in the total number of predicted petroleum deposits. We take the minimum number of predicted existing petroleum deposits by a single model to achieve this saturated value as the threshold value. We keep only those models that can predict at least the threshold number of petroleum deposits in the testing region.

### n-Voting over Results of ML

After filtering the best-performing model outputs that can predict at least a threshold number of petroleum deposits, we can get only top-performing model outputs. Each of the filtered-out models can predict a number of existing petroleum deposits. These models also output some more points as potential petroleum deposits that are yet to be discovered. Now, since each of the best-performing models can predict some (not all) of the existing petroleum deposits, the potential petroleum deposits need to be verified by other best-performing models. To perform this verification finally, we use an n-voting system. Here, if there are n best-performing models, we can get 1 to n votes for each of the potential petroleum deposits. Accordingly, in our n-voting system, if any point is selected by at least n votes, then the point is selected as a potential petroleum deposit. In this way, we filter out some areas that are highly probable to have petroleum deposits. To do this, we keep a count of the number of votes in the n-voting system. Using this count, we get more probable petroleum deposits in our study area, which we present using a heatmap. Here, since each point is selected only if the point gets predicted by at least n different models, we name the method as nAND-ing.

### Graph-based Analysis for Maximum Coverage with Minimum Number of Resulting Output Models

**Figure 3:**
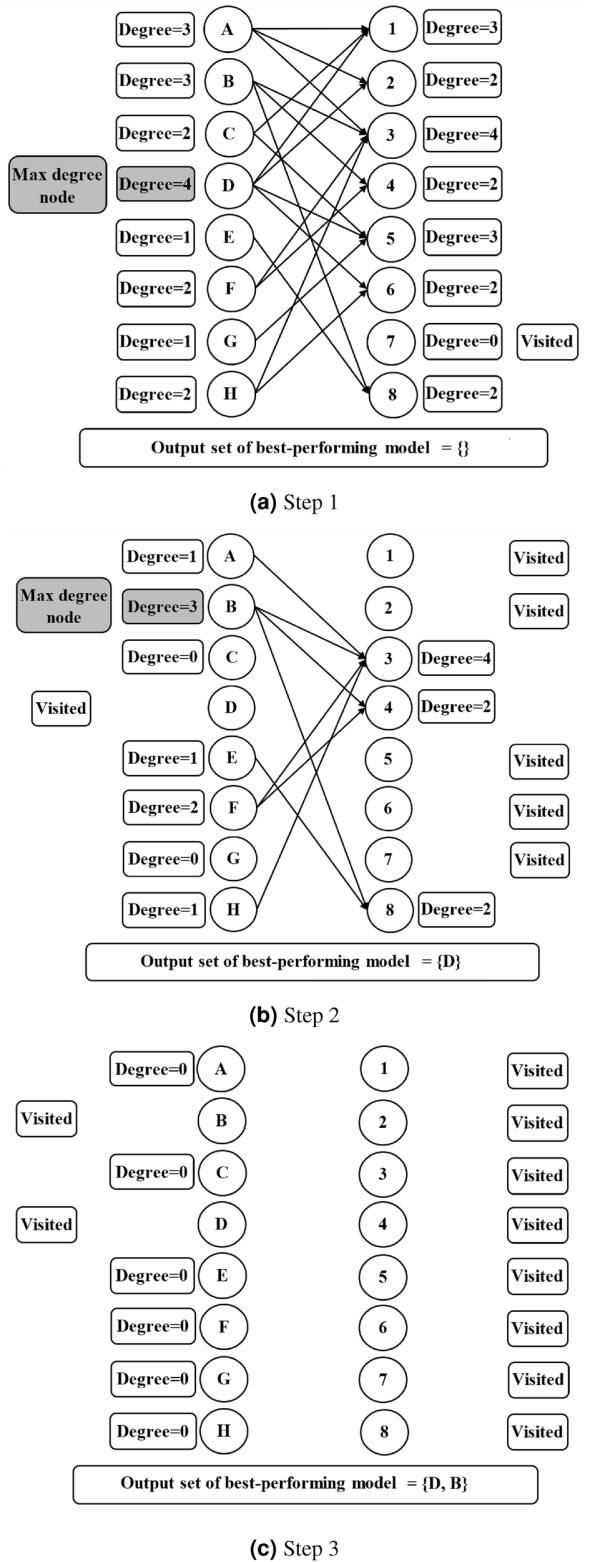
Steps of optimal model coverage following our proposed algorithm based on graph-based analysis.

At the final stage of our study, we attempt to develop a generalized method to cover all petroleum deposits with a minimum number of models, i.e., rows in the matrix. To do so, we build a bipartite graph^47^ consisting of the best-performing models in one part (as “Part A”) and existing petroleum deposits in another part (as “Part B”). We put an edge from a vertex in Part A to a vertex in Part B, if the corresponding best-performing model in Part A can detect the corresponding petroleum deposit in Part B. Then, we try to find an algorithm that will find the minimum vertex set from Part A that has at least one edge to cover all the vertices of Part B, i.e., all the petroleum deposits in Part B except those that do not have any edges. This turns into a “vertex cover in hypergraphs” problem over the bipartite graph^48^. We build Algorithm 1 as our heuristic-based greedy algorithm to cover all petroleum deposits (Part B) with a minimum number of vertices in Part A. Figure 3 shows an example case of our Algorithm 1 portraying steps of the algorithm.

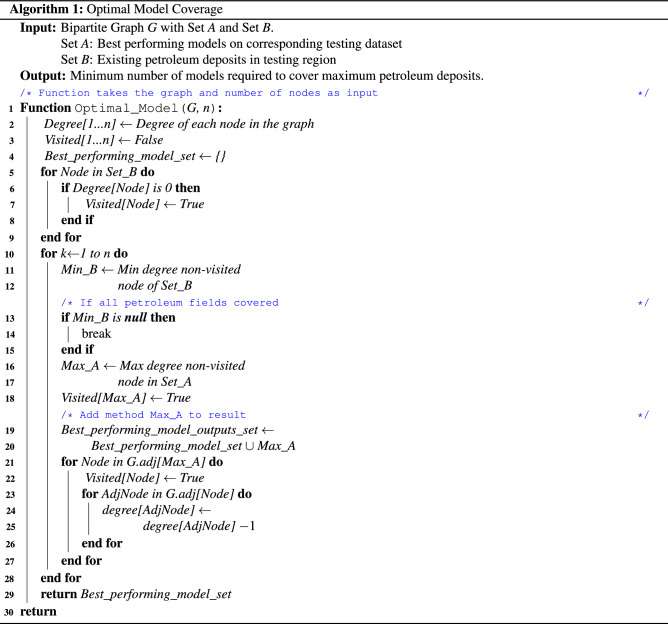



## Experimental Setup

For the purpose of our experimentation, we first need to build our training datasets. To do that, we gather GRACE satellite data. As GRACE gets operated jointly by the Jet Propulsion Laboratory (JPL) from NASA, the Center for Space Research from the University of Texas, Austin (UTCSR), and the German Space Research Center (GFZ)^49^, each of these three institutions processes the raw data from GRACE and releases them as a different source of the data. They also release different versions of the processed data. This data is found from Physical Oceanography Distributed Active Archive Center (podaac)^50^. Note that, in spite of the data being free at this site, one needs to open a free account there to download the data. We take the GRACE data from here by opening a free account. We have used the release 05 (RL-05) data of GRACE from the site. Accordingly, in our experiments, we consider all the different sources of GRACE data of RL-05. These include JPL GSM90, GFZ GSM90, UTCSR GSM60, and UTCSR GSM96.

The data sources provide us with the monthly average of the spherical harmonic coefficients of the earth. Using these spherical harmonic coefficients, we calculate the GGT components (corresponding calculations are given in the Appendix). As discussed earlier, GGT can be calculated only for specific points on earth. Therefore, in our study, we divide the earth by a $0.1^\circ \times 0.1^\circ$ degree grid. Then we calculate GGT for each point on the grid.

In our study area, $0.1^\circ$ degree on earth measures different lengths in kilometers at different locations. For example, at the north of our training region, $0.1^\circ$ degree is equivalent to approximately 6.7km along the east-west direction. On the other hand, at the south of our training region, $0.1^\circ$ degree is equivalent to approximately 10.7km along the east-west direction. Note that, along the north-south direction, $0.1^\circ$ degree is equivalent to approximately 11km for our study area. Besides, as calculating GGT components is a time-consuming job, we try to overcome the repetition of the calculations of GGTs by calculating the GGT components of all the points over our area of study in a $0.1^\circ \times 0.1^\circ$ grid and saving them in computer storage. We take the values from the storage during experimentation mimicking the notion of dynamic programming.

**Figure 4:**
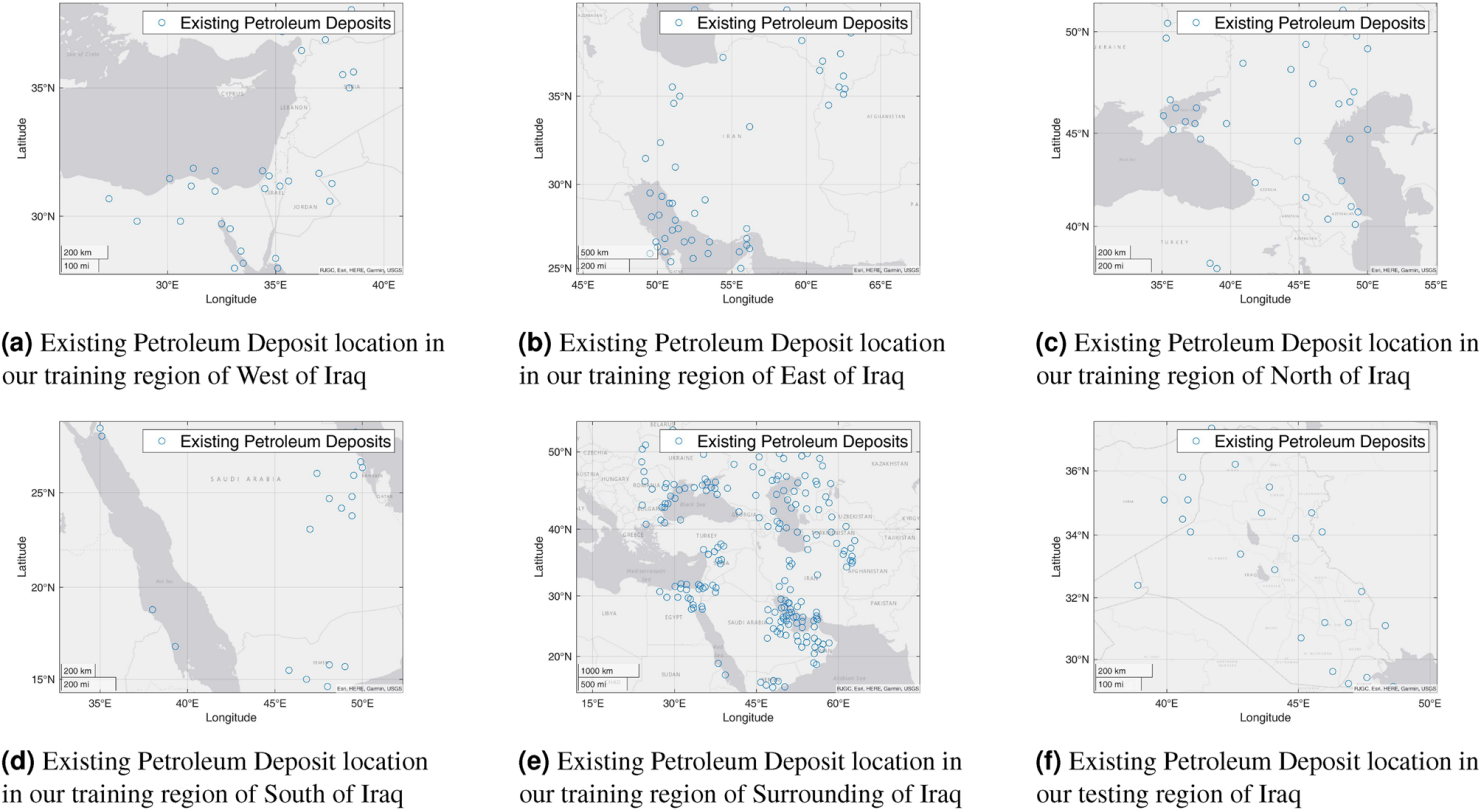
Existing petroleum deposit locations of our training and testing regions (the figures are created using MATLAB 2021a^51^).

**Table 2:** The density of giant, mega-giant, and super-giant petroleum deposits in our training and testing regions.

Region	# of Giant, Mega Giant, and Super Giant Petroleum Deposits	Area (in Sq Degree)	A Petroleum Deposit Appears within an Area (in Sq Degree)
Whole World	1273	64800	50.90
Left of Iraq	31	225	7.26
Right of Iraq	50	225	4.50
Top of Iraq	34	225	6.62
Bottom of Iraq	21	225	10.71
Surround of Iraq	196	1461.68	7.46
Iraq itself	26	82.32	3.17

**Figure 5:**
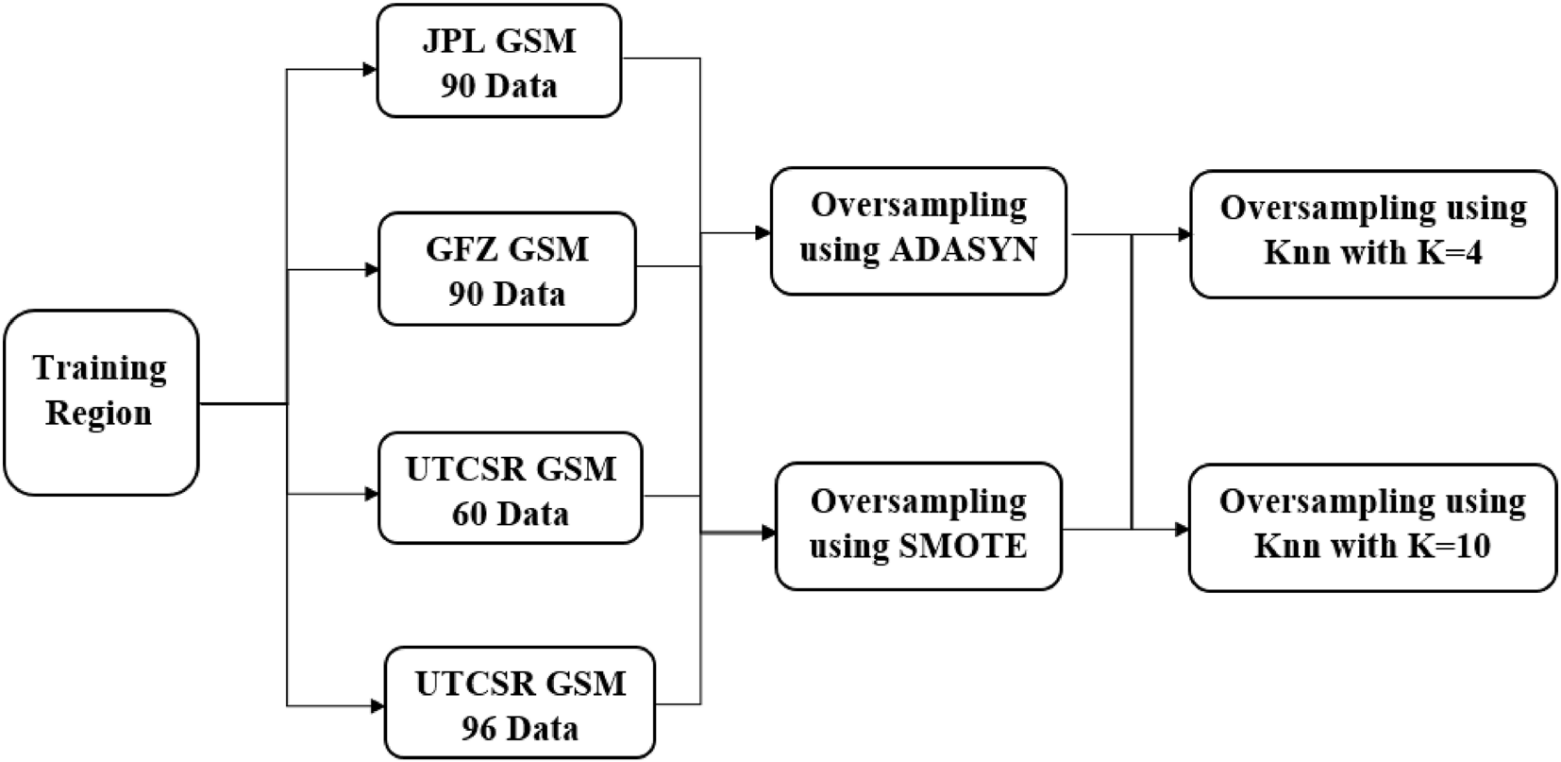
The process of building our training dataset.

As mentioned earlier, we have five training and one testing regions within our area of study. Accordingly, we build training datasets for all the training regions and testing datasets for the testing region. To do this, we calculate the GGT for each point of these regions. After calculating the GGT components of these regions, we need to consider the locations of the giant, supergiant, and mega-giant petroleum deposits within those regions to prepare our dataset. We collect the locations of the giant, supergiant, and mega-giant petroleum deposits all over the world from the Peace Research Institute Oslo (PRIO)^9^. From the list, for each of the regions under our study, we take the locations of existing petroleum deposits. Table 2 presents the number of petroleum deposits in our training and testing regions as well as the whole world. We can see the area in square degrees within which one petroleum deposit appears for different regions from the Table. Figure 4 shows the locations of the existing petroleum deposits in our training and testing regions. Now, for each point of the grid in a single region, we take all six GGT components and label them as ‘0’ (false) if there is no petroleum deposit. Besides, we label them as ‘1’ (true) if that point has a petroleum deposit. We follow the same procedure for all the training and testing regions. Figure 5 shows this procedure based on which we make our training and testing datasets for the training and testing regions.

As we take GRACE data from four different data sources, for each training region, we get four sets of data. Here, the number of true points inside the data is very less compared to that of false points as the number of existing petroleum deposits is much smaller than the number of non-petroleum points. Therefore, we apply oversampling techniques to the dataset from each data source to make the dataset a balanced one. We used two standard oversampling methods on each dataset. Those methods are SMOTE^45^ and ADASYN^46^. Note that, each oversampling method is applied to each data source two times with a KNN - 1) where the value of K is 4, and 2) where the value of K is 10. As a result, each data source is oversampled four times. Therefore, for each imbalanced dataset from a single data source, we get four oversampled balanced datasets as follows.

- Oversampled using SMOTE with KNN where K = 4,
- Oversampled using SMOTE with KNN where K = 10,
- Oversampled using ADASYN with KNN where K = 4, and
- Oversampled using ADASYN with KNN where K = 10.

Since there are four different data sources, we get a total of 16 oversampled training datasets in each region. Figure 5 shows the creation process of the 16 datasets from one single region. Finally, as we have five training regions, and each one has 16 balanced oversampled training datasets, we get a total of 80 balanced oversampled training datasets. The testing dataset is built in the same way as training datasets except performing the oversampling part. Note that, initially we explore with KNN where K = 4, 5, 6, 7, 8, 9, and 10. However, we do not find any notable performance change between the intermediate values of 4 and 10. Therefore, we continue our further study with only the values of 4 and 10.

## Results and Findings

We prepare 80 training datasets following the process mentioned in the experimental setup. We apply 28 different machine learning (ML) techniques over all the training datasets pertinent to different training regions. To do this, we use a well-known machine-learning tool named Auto-WEKA^52^. While training with the 80 datasets using 28 different ML techniques, we used the standard 10-fold cross-validation for our datasets, as 10-fold cross-validation is a widely used training-validating method nowadays. We find different outcomes from different ML techniques for the same dataset. We take the best-performing ML technique for each training dataset separately. We find that the best-performing ML techniques perform very well for their respective training regions. We have shown the precision, recall, F1 score, and accuracy of the 16 best-performing training datasets for their respective training regions in Table 3. We save each of the models found from the best-performing methods separately. Then, we apply those best-performing models to corresponding testing datasets. This time the performance is not as good as we have found during the training. Table 3 also shows the precision, recall, F1 score, and accuracy of the best-performing models when we apply them to the corresponding testing datasets.

**Table 3:** Evaluation metrics for trained models.

Training Region	Data Source	Oversampling Technique	KNN	Training Time (min)	Training					Testing				
					Precision	Recall	F-Measure	ROC Area	Accuracy	Precision	Recall	F-Measure	ROC Area	Accuracy
RIGHT	GFZ	SMOTE	10	150	1.000	1.000	1.000	1.000	1.000	0.994	0.672	0.801	0.584	0.672
RIGHT	UTCSR 60	SMOTE	10	15	0.999	0.999	0.999	1.000	0.999	0.994	0.649	0.784	0.507	0.649
RIGHT	JPL	SMOTE	10	15	0.999	0.999	0.999	1.000	0.999	0.994	0.778	0.872	0.599	0.778
Surrounding	JPL	ADASYN	10	1920	1.000	1.000	1.000	1.000	1.000	0.994	0.689	0.813	0.548	0.689
RIGHT	JPL	ADASYN	10	15	1.000	1.000	1.000	1.000	1.000	0.994	0.752	0.856	0.569	0.752
TOP	JPL	SMOTE	10	15	1.000	1.000	1.000	1.000	1.000	0.995	0.829	0.904	0.656	0.829
RIGHT	GFZ	ADASYN	10	15	1.000	1.000	1.000	1.000	1.000	0.994	0.795	0.883	0.577	0.795
RIGHT	GFZ	SMOTE	10	15	0.999	0.999	0.999	1.001	0.999	0.994	0.835	0.907	0.580	0.835
RIGHT	UTCSR 60	ADASYN	10	15	0.999	0.999	0.999	1.000	0.999	0.994	0.705	0.824	0.465	0.705
RIGHT	UTCSR 96	SMOTE	10	15	0.999	0.999	0.999	1.000	0.999	0.994	0.816	0.896	0.455	0.816
Surrounding	JPL	SMOTE	10	120	1.000	1.000	1.000	1.000	1.000	0.994	0.856	0.920	0.599	0.856
TOP	GFZ	ADASYN	10	15	1.000	1.000	1.000	1.000	1.000	0.994	0.818	0.897	0.549	0.818
TOP	JPL	ADASYN	10	15	1.000	1.000	1.000	1.000	1.000	0.994	0.850	0.916	0.645	0.850
Bottom	JPL	ADASYN	10	15	0.999	0.999	0.999	1.000	0.999	0.994	0.776	0.871	0.502	0.776
RIGHT	UTCSR 96	ADASYN	10	15	0.999	0.999	0.999	1.000	0.999	0.994	0.822	0.900	0.473	0.822
Surrounding	GFZ	ADASYN	10	1920	1.000	1.000	1.000	1.000	1.000	0.994	0.739	0.847	0.533	0.739

**Table 4:** Matrix with the best-performing models (among 80 models) and their predicted petroleum deposits in the testing region.

Sl	Models	Petroleum Field No																										
		1	2	3	4	5	6	7	8	9	10	11	12	13	14	15	16	17	18	19	20	21	22	23	24	25	26	Count
1	RIGHT_GFZ_SMOTE_10_150min	0	0	0	0	0	0	0	0	1	0	1	0	1	0	1	0	1	0	1	1	1	1	0	1	1	1	12
2	RIGHT_UTCSR 60_SMOTE_10	0	0	1	0	0	0	1	0	1	1	0	1	1	0	0	1	1	0	1	1	0	1	0	0	0	0	11
3	RIGHT_JPL_SMOTE_10	0	0	0	0	0	1	0	0	0	0	1	0	0	0	1	0	1	0	1	0	0	1	1	1	1	1	10
4	Surrounding_JPL_ADASYN_10_1920min	0	0	0	0	0	0	0	0	1	0	1	0	1	0	1	0	1	1	0	0	1	0	1	1	1	0	10
5	RIGHT_JPL_ADASYN_10	0	0	0	0	0	1	0	0	1	0	1	0	0	0	1	0	1	0	0	0	0	1	0	1	1	1	9
6	TOP_JPL_SMOTE_10	0	0	0	0	0	1	0	1	1	0	0	1	0	0	0	0	1	1	0	0	1	0	0	0	1	1	9
7	RIGHT_GFZ_ADASYN_10	0	0	0	0	0	1	0	0	1	0	1	0	0	0	0	0	1	0	0	1	1	0	0	1	1	0	8
8	RIGHT_GFZ_SMOTE_10	0	0	0	0	0	0	0	0	1	0	1	0	0	0	0	0	1	0	0	0	1	1	0	1	1	0	7
9	RIGHT_UTCSR 60_ADASYN_10	0	0	1	0	0	0	1	1	0	1	1	1	0	0	0	0	0	0	0	0	0	1	0	0	0	0	7
10	RIGHT_UTCSR 96_SMOTE_10	0	0	0	0	0	0	0	0	0	1	1	0	0	1	0	0	0	0	0	0	0	1	0	1	1	1	7
11	Surrounding_JPL_SMOTE_10_120min	0	0	0	0	0	1	0	0	1	0	1	0	0	0	1	0	0	0	0	0	0	0	0	1	1	1	7
12	TOP_GFZ_ADASYN_10	0	0	0	0	1	0	1	1	0	0	0	1	0	0	0	0	1	0	0	0	0	0	0	0	1	1	7
13	TOP_JPL_ADASYN_10	0	0	0	0	0	1	0	1	0	0	0	1	0	0	0	0	1	0	0	0	1	0	0	0	1	1	7
14	Bottom_JPL_ADASYN_10	0	1	1	1	0	0	0	0	0	0	0	0	0	1	0	1	0	0	0	1	0	0	0	0	0	0	6
15	RIGHT_UTCSR 96_ADASYN_10	0	0	0	0	0	0	0	0	0	0	1	0	0	1	0	0	0	0	0	0	0	1	0	1	1	1	6
16	Surrounding_GFZ_ADASYN_10_1920min	0	0	0	0	0	0	0	0	1	0	0	0	1	0	1	0	1	0	0	0	1	0	0	0	1	0	6
	Total number of times the field detected	0	1	3	1	2	6	3	5	9	3	10	6	4	3	6	2	11	2	3	4	7	8	2	9	14	10	

**Figure 6:**
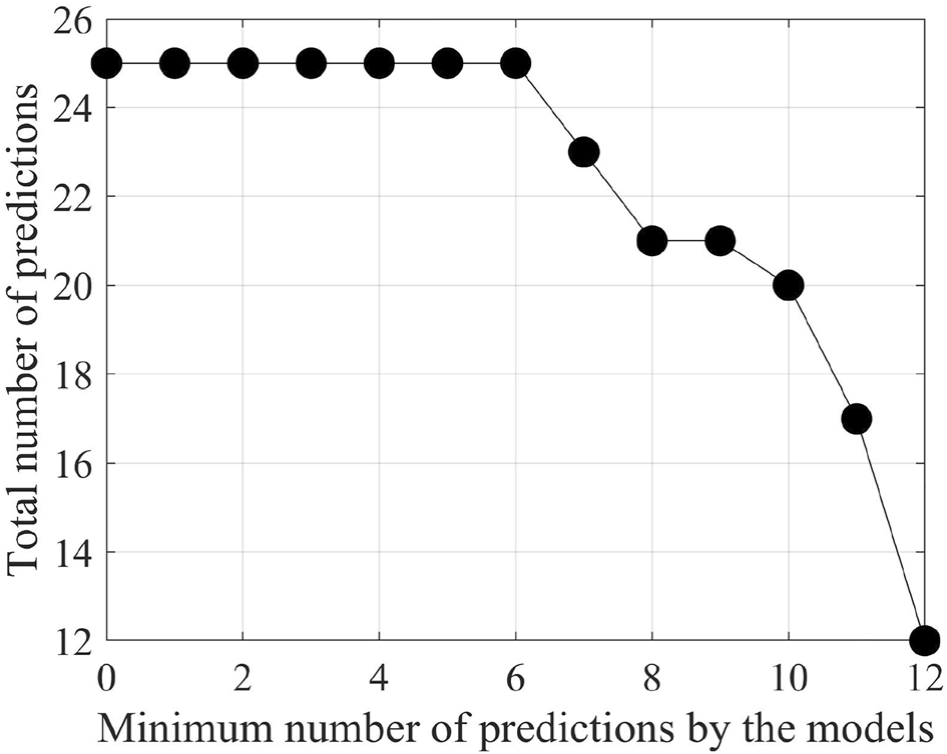
The process of finding the threshold.

It is important to keep in mind that we have 26 existing petroleum deposits in each of our testing datasets. As the testing region is the same for all the testing datasets, therefore, the 26 existing petroleum deposits are the same for all the testing datasets. As per our findings, none of the best-performing models can predict all of the 26 deposits. In fact, one of the resulting models can predict a maximum of 12 existing deposits. Besides, for different models, the predicted petroleum deposits vary. Similarly, other resulting models from some other different training datasets can predict less than 12 (eleven, ten, or fewer) petroleum deposits in the testing datasets. In most cases, the predicted petroleum deposits vary for different models. To analyze which petroleum deposit in the testing datasets gets predicted by which model and which petroleum deposits are predicted most of the time, we prepare a matrix. In the matrix, we take 26 columns for all 26 petroleum deposits in the testing region. We take the output of all the best-performing models on the corresponding testing datasets from the 80 training datasets as the rows of the matrix. Then, we sort rows of the matrix in descending order based on the total number of predicted petroleum deposits in the testing region. We find that one of the petroleum deposits in the testing region can not be predicted by any of the models. Besides, some models detect only a few (two or three) existing deposits. Therefore, we keep only those resulting models, which can predict at least six petroleum deposits in the testing region. Table 4 shows the matrix with the best-performing models that can predict at least six of the petroleum deposits in the testing region. We take the threshold value as six, as decreasing the threshold value further does not result in any increase in the total number of predicted petroleum deposits. Accordingly, we consider six as the value of reaching the saturation point in the process of predicting the maximum number of petroleum deposits in the testing region. Therefore, we adopt six as the threshold value. Figure 6 shows how the threshold value gets selected through the process of getting the maximum number of predicted petroleum deposits saturated.

**Figure 7:**
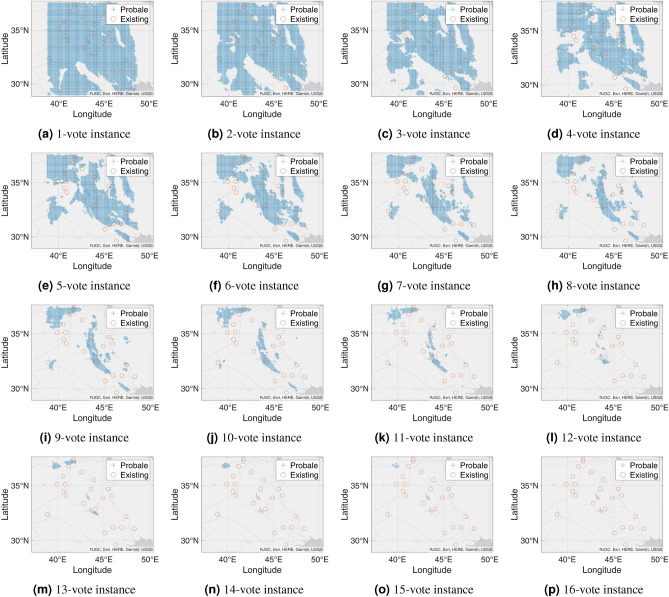
Probable petroleum deposits based on the existence of 1 to 16 votes over our 16 best-performing models from the matrix-based analysis (the figures are created using MATLAB 2021a^51^).

**Figure 8:**
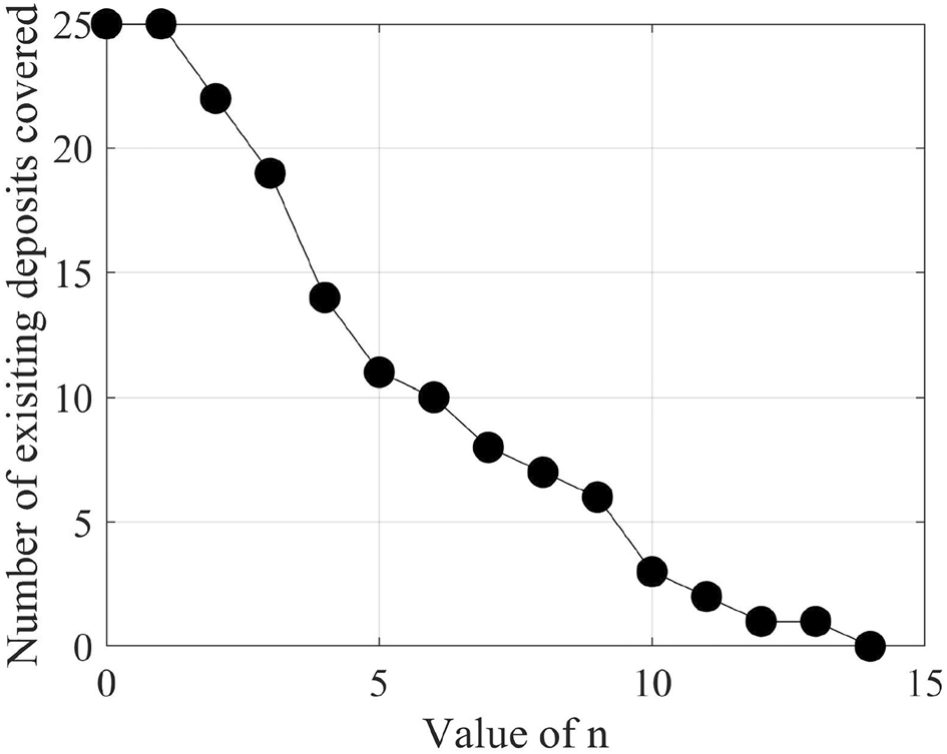
Change in the number of existing petroleum deposits covered by n-vote instances with an increasing value of n.

**Figure 9:**
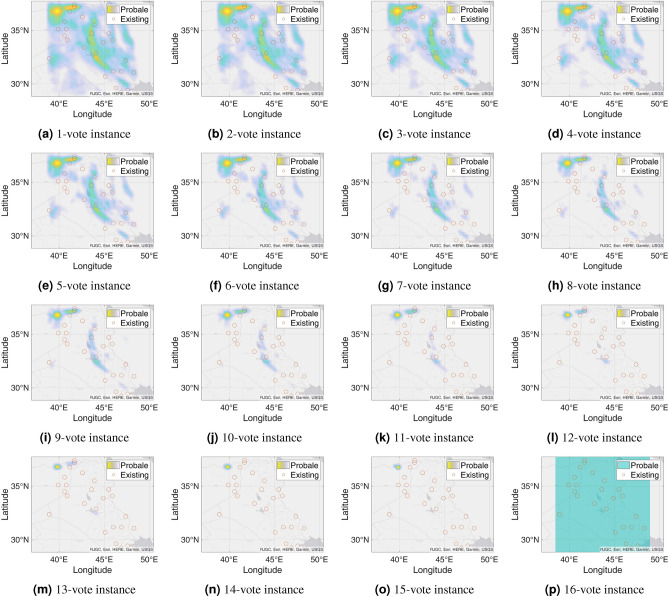
Probable petroleum deposits based on the frequency of 1 to 16 votes over our 16 best-performing models from the matrix-based analysis (the figures are created using MATLAB 2021a^51^).

### n-Voting over Results of ML Methods

After filtering the best-performing models that can predict at least six petroleum deposits in the testing datasets, we get a total of 16 best-performing models as shown in Table 4. Each of these 16 models can predict a number of existing petroleum deposits. On the other hand, these models also indicate some more points as probable undiscovered petroleum deposits. Since each of the best-performing models can predict some of the existing petroleum deposits, the indications of probable undiscovered petroleum deposits need to be verified by other best-performing models. To enable the verification, we use an n-voting mechanism. Here, as there are 16 best-performing models available, we can get 1 to 16 votes for verifying a probable petroleum deposit. Thus, as per the n-voting mechanism, if any point is selected by n votes, then the point is considered to be a verified probable undiscovered petroleum deposit. Figure 7 shows the outcome of the n-voting mechanism for n = 1 to 16, on our testing datasets. The best case in Fig. 7 (i.e., Fig. 7a or the 1-vote instance) covers 25 out of the existing 26 deposits. In the case of 2-vote instance (i.e., Fig. 7b), the coverage decreases to 22 out of the existing 26 deposits, and so on. Figure 8 presents the scenario. From the subfigures of Fig. 7, we can see that a large portion of our study area is probable for petroleum deposits with a small value of n. However, as we are interested in areas that are highly probable to have petroleum deposits, we keep a count of the number of votes in the n-voting mechanism. Using these increased values of n, we get the more probable petroleum deposits in our study area. We build heatmaps using these counts as shown in Fig. 9.

**Figure 10:**
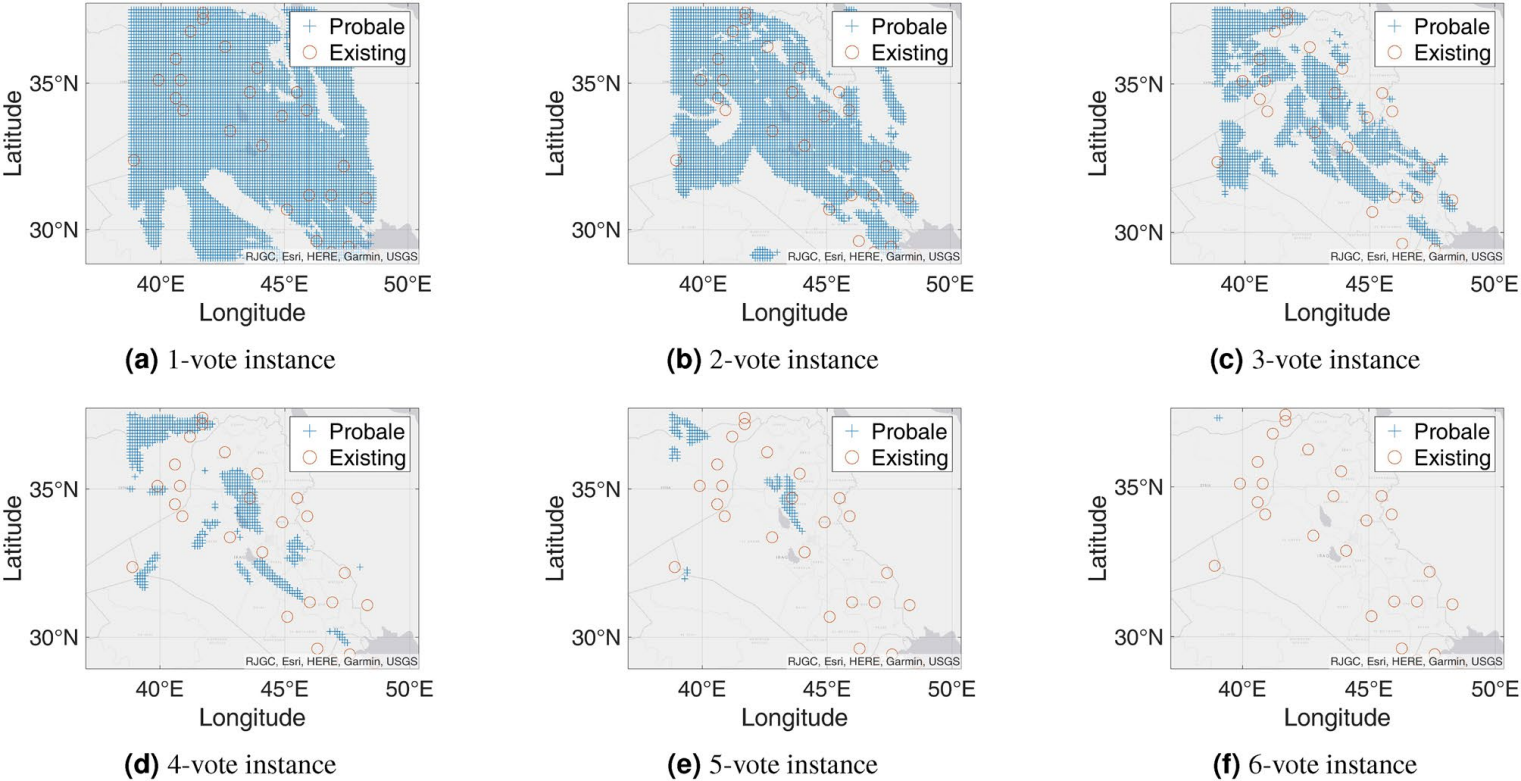
Probable petroleum deposits based on the existence of 1 to 6 votes over our 6 best-performing models found from the graph-based analysis (the figures are created using MATLAB 2021a^51^).

### Graph-based Analysis for Maximum Coverage with Minimum Number of Resulting Model Outputs

For the purpose of covering the maximum existing deposits in our testing region using a minimum number of models, we build a bipartite graph where the set *A* contains all the best-performing models. Besides, we take the set *B*, which contains all the existing petroleum deposits of the testing region. We put an edge from a node in the set *A* to a node in the set *B* when a best-performing model from set *A* predicts a petroleum deposit from set *B*. Algorithm 1 covers the maximum number of elements from set *B* using the minimum number of elements from set *A*. We get a set of six best-performing modes that cover a total of 25 petroleum deposits. As mentioned earlier, out of 26 deposits, one deposit is not predicted by any of the models. The predicted deposits are shown in Table 5. Besides, we show performances of the top 16 trained models in Table 3. Among the performance metrics, we consider the ‘Recall’ values to be the most crucial, as this metric denotes the proportion of properly identified petroleum deposits among the existing ones.

**Table 5:** Matrix with the minimum number of best-performing models that can detect the maximum number of deposits in the testing region using the models that can detect at least six of the existing petroleum deposits.

Models	Petroleum Field No																										
	1	2	3	4	5	6	7	8	9	10	11	12	13	14	15	16	17	18	19	20	21	22	23	24	25	26	Count
RIGHT_GFZ_SMOTE_10_150min	0	0	0	0	0	0	0	0	1	0	1	0	1	0	1	0	1	0	1	1	1	1	0	1	1	1	12
RIGHT_UTCSR 60_SMOTE_10	0	0	1	0	0	0	1	0	1	1	0	1	1	0	0	1	1	0	1	1	0	1	0	0	0	0	11
Bottom_JPL_ADASYN_10	0	1	1	1	0	0	0	0	0	0	0	0	0	1	0	1	0	0	0	1	0	0	0	0	0	0	6
TOP_JPL_SMOTE_10	0	0	0	0	0	1	0	1	1	0	0	1	0	0	0	0	1	1	0	0	1	0	0	0	1	1	9
TOP_GFZ_ADASYN_10	0	0	0	0	1	0	1	1	0	0	0	1	0	0	0	0	1	0	0	0	0	0	0	0	1	1	7
RIGHT_JPL_SMOTE_10	0	0	0	0	0	1	0	0	0	0	1	0	0	0	1	0	1	0	1	0	0	1	1	1	1	1	10
Total	0	1	2	1	1	2	2	2	3	1	2	3	2	1	2	2	5	1	3	3	2	3	1	2	4	4	

**Figure 11:**
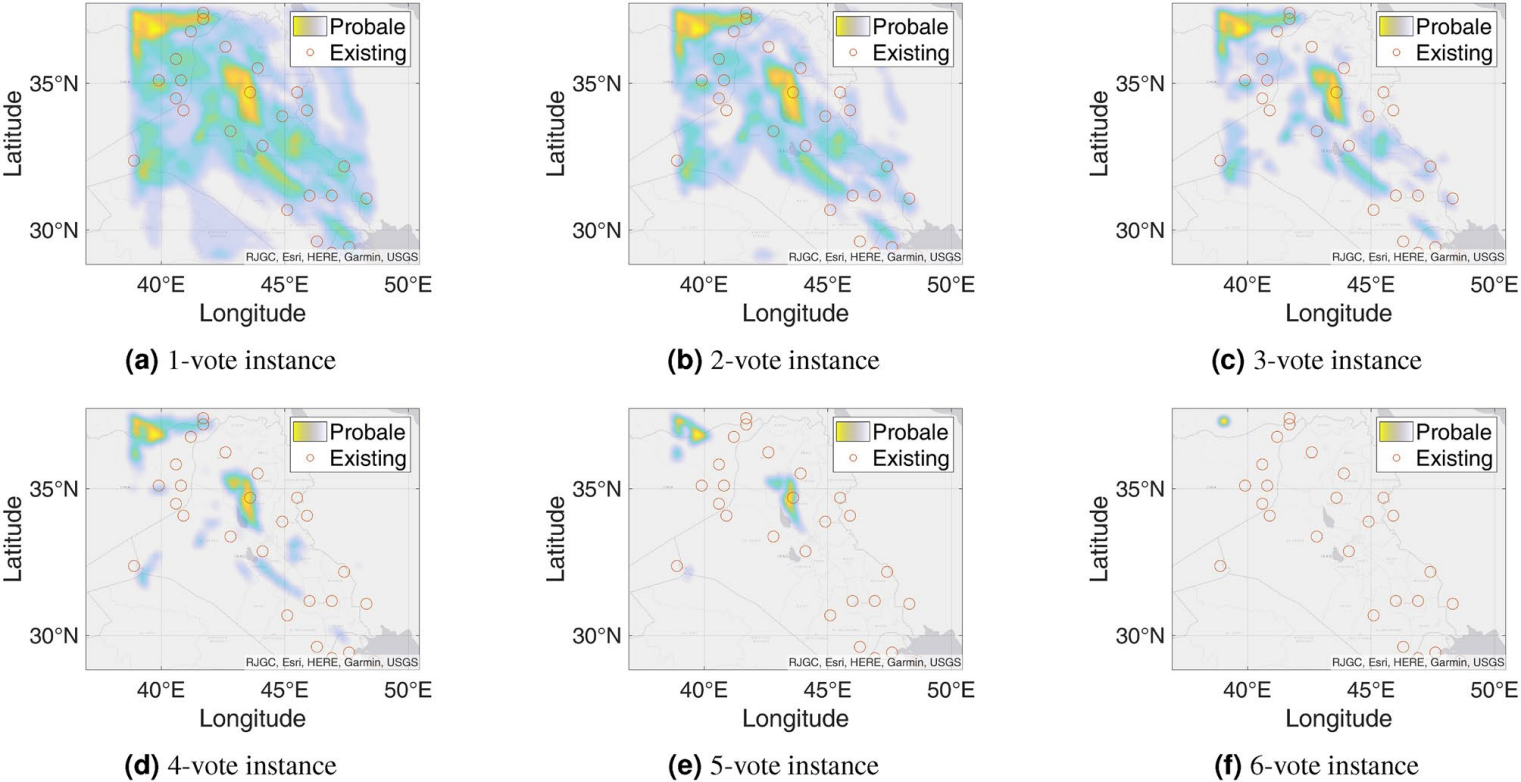
Probable petroleum deposits based on the frequency of 1 to 6 votes over our six best-performing models found from the graph-based analysis (the figures are created using MATLAB 2021a^51^).

After completing the graph-based analysis, we find six best-performing models that resulted in the minimum number of models needed to predict the maximum possible deposits. On the basis of the found six models, Fig. 10 shows binary decision outcomes based on 1-to-6 voting. Besides, Fig. 11 shows corresponding heatmaps for the 1-to-6 voting. The n-vote binary decision outcomes are obtained in the following way. From the bipartite graph, we apply the n-voting method to the six best-performing models that unitedly identify the 25 petroleum deposits. This time, we apply 1 to 6 votes as there are only six best-performing resulting models. We apply the best-performing models to our testing datasets and get indications of some probable undiscovered petroleum deposits when any point in our testing datasets gets predicted as a probable petroleum deposit at least n times in an n-voting system. If a point is not predicted for at least n times, we indicate that point as a non-petroleum location.

Figure 10a presents the result obtained from 1-voting. Here, we can see a huge number of points indicated as probable petroleum deposits. This happens as any of the best-performing models predicts any point, it gets selected as a potential petroleum deposit. In other words, this result is a union of all six best-performing models’ outputs.

In the case of 2-voting, the result presents fewer predicted points than 1-voting as shown in Fig. 10b. Here, a point is considered a probable petroleum deposit if that point is predicted by at least two of the best-performing models. If a point cannot be detected by at least two best-performing models, then we mark that point as a non-petroleum location. The results contain a large number of predicted points. Subsequently, we explore 3-voting. This time we take a point as a probable petroleum deposit if that point is predicted by at least three best-performing models. Any point that does not comply with this condition is marked as a non-petroleum point. Figure 10c presents the output of the 3-voting. We can see that this figure predicts fewer points as probable petroleum deposits than 2-voting.

Later, we explore 4-voting and 5-voting in a similar way. The results are shown in Fig. 10d,e respectively. The 4-voting gives us a concise view of probable petroleum deposits, which are comprised of a few regions in addition to having some single discrete points. Besides, the 5-voting gives us a clear view of the locations of new probable petroleum deposits. In the outcome of the 5-voting, we can see that the new probable petroleum deposits converge towards two locations.

Finally, we explore 6-voting. Here, we take an intersection over the outputs of all the six best-performing models. Figure 10f presents the outcome of the 6-voting. This time our system can predict only a few points as probable petroleum deposits. As the points are predicted by all the best-performing models, these points are highly probable to have some petroleum deposits. Thus, the probability of a new petroleum deposit is very high at these predicted locations. However, as this figure shows the outcome of intersection over all the best-performing models resulting in only a few predicted locations, we can understand that some other real deposits may remain undiscovered due to taking the intersection operation.

We present another type of result, which is comprised of heatmaps. Unlike binary decision results, heatmap results focus not only on the presence of the petroleum deposit but also on the frequency of prediction of each point. The point that gets predicted more times by the best-performing models is more probable as a new petroleum deposit. We represent the result using heatmaps in Fig. 11. Here, Fig. 11a shows the 1-voting heatmap results. Similar to the binary decision system, we take a point if any of the six best-performing models can predict the point. If any point is predicted by more than one best-performing model, then we increase the frequency of that point according to their frequency of prediction. Note that, we can see that approximately the whole image is predicted as petroleum deposits for a 1-vote heatmap. However, some points are indicated with dark color as these points have high frequencies and are most probable as petroleum deposits. In other words, this is the superposition counting for all points found from all the six best-performing models.

Similar to the previous case, we explore 2-voting through a heatmap representation and get Fig. 11b. Here, we consider a point as a potential petroleum deposit if at least two best-performing models can predict that point. If more than two best-performing models predict any point, we increase the frequency of that point based on the number of predicting best-performing models. Here, we can see a smaller number of shaded places in this picture than that we have found for 1-voting. Using the same procedure, we further explore the 3-voting heatmap as shown in Fig. 11c. Here, similar to the previous case of exploring the 3-voting, the 3-voting heatmap shows fewer probable points.

In a similar way, we get heatmaps for 4-voting and 5-voting as shown in Fig. 11d, e. From the 4-voting heatmap, we can see that the probable new petroleum deposits are divided into smaller regions. Besides, from the 5-voting heatmap, we can see that only two specific regions have new probable petroleum deposits. Finally, we get a heatmap from 6-voting as shown in Fig. 11f. This image shows only one location as the new probable petroleum deposit. As this point is predicted by all the best-performing models, there is a very high chance of having a petroleum deposit here. However, from the 5-voting heatmap, we see one more region is probable for a new deposit compared to the outcome found from 6-voting. Note that, as the 6-voting heatmap realizes predictions of all the best-performing models altogether, some other probable petroleum deposits might not get predicted by the 6-voting prediction.

**Table 6:** Matrix with the best-performing models (among 80 models) and their predicted petroleum deposits in the testing region for the new test dataset from Harvard.

Models	Petroleum Field No																																																									
	1	2	3	4	5	6	7	8	9	10	11	12	13	14	15	16	17	18	19	20	21	22	23	24	25	26	27	28	29	30	31	32	33	34	35	36	37	38	39	40	41	42	43	44	45	46	47	48	49	50	51	52	53	54	55	56	57	Count
RIGHT_GFZ ADASYN_10	0	0	0	1	1	1	1	1	1	1	0	0	0	0	0	0	0	0	0	0	0	1	1	1	0	0	0	1	0	0	0	0	1	1	0	1	1	1	1	1	0	0	0	1	0	0	0	0	1	1	0	1	1	1	0	0	1	25
Surrounding_GFZ ADASYN_10_1920min	0	0	0	1	1	1	0	0	1	1	0	0	0	0	0	0	0	0	0	0	0	0	0	0	0	0	0	1	1	0	1	0	0	1	0	1	1	0	0	1	1	1	1	1	1	1	0	1	1	1	0	1	1	1	0	0	1	25
Surrounding_JPL ADASYN_10_1920min	0	0	0	0	1	1	1	1	1	1	0	0	0	0	0	0	0	0	0	0	0	0	0	0	0	1	0	1	1	1	0	0	1	1	1	0	0	0	0	1	1	1	0	1	1	1	0	1	1	1	0	1	1	1	0	0	0	25
RIGHT_GFZ SMOTE_10_150min	0	0	0	1	1	0	0	0	1	1	0	0	0	0	0	0	0	0	0	0	0	0	0	0	0	0	0	0	1	1	1	0	1	1	1	1	0	0	0	1	1	1	1	1	1	1	0	1	0	0	0	0	1	1	0	1	1	23
RIGHT_UTCSR 60 SMOTE_10	1	1	0	0	0	0	0	0	1	1	0	0	0	0	0	1	0	1	1	1	1	1	0	0	1	1	0	1	1	1	1	0	1	0	1	1	0	1	0	1	0	0	0	0	0	1	0	0	1	0	0	0	0	0	0	0	0	23
RIGHT_JP ADASYN_10	0	0	1	0	0	0	0	0	1	1	0	0	0	0	0	0	0	0	0	1	0	1	1	0	0	1	0	1	1	1	0	0	1	0	1	0	0	1	1	0	0	0	0	0	0	1	0	1	0	0	0	0	1	1	1	1	1	21
RIGHT_JPL SMOTE_10	0	0	1	1	1	0	1	0	1	1	0	0	0	0	0	1	0	0	0	1	0	1	1	0	0	0	0	0	0	0	0	0	0	0	0	0	0	1	1	1	1	1	0	1	1	0	0	1	0	0	0	0	1	0	0	1	1	21
TOP_JPL SMOTE_10	0	0	0	0	0	0	0	0	0	0	0	0	0	0	0	0	0	0	0	1	0	1	1	1	0	1	1	1	1	0	1	1	1	1	1	0	0	0	0	0	0	0	0	0	0	0	0	0	0	0	1	1	1	1	0	0	0	17
Surrounding_GFZ SMOTE_10_120min	0	0	0	0	0	0	0	0	1	1	0	0	0	0	0	0	0	0	0	0	0	0	0	0	1	0	1	0	0	0	1	1	0	1	1	1	1	1	1	0	0	0	0	0	0	0	0	0	1	1	0	0	0	0	0	0	1	15
Surrounding_UTCSR 96 SMOTE_10_1000min	0	0	0	0	0	0	1	1	0	0	1	1	1	1	0	0	0	0	0	0	0	0	0	0	0	0	0	0	0	1	1	0	0	1	1	0	0	0	0	0	0	0	0	1	1	0	0	1	0	0	0	0	0	0	0	1	1	15
RIGHT_GFZ SMOTE_10	0	0	0	0	0	1	0	0	1	1	0	0	0	0	0	0	0	0	0	0	0	0	0	1	0	0	0	0	0	0	1	0	1	0	0	1	1	1	0	1	0	0	0	0	0	0	0	0	1	1	0	0	1	1	0	0	0	14
Surrounding_GFZ ADASYN_4_120min	0	0	0	0	0	0	0	0	1	1	0	0	0	0	0	0	0	0	0	0	0	0	0	0	1	0	0	0	0	0	0	1	0	1	0	1	1	0	1	1	0	0	1	0	0	1	0	0	1	1	0	1	0	0	0	0	0	14
Surrounding_JPL SMOTE_10_120min	0	0	0	0	0	1	0	0	1	1	0	0	0	0	0	0	0	0	0	1	0	1	1	0	1	0	0	1	0	0	0	0	0	1	0	0	0	0	0	0	0	0	0	1	0	0	0	1	0	0	0	0	1	0	0	0	1	13
RIGHT_UTCSR 60 ADASYN_10	0	1	0	0	0	0	0	0	1	0	0	0	0	1	1	0	1	1	1	0	1	0	0	0	1	0	1	0	0	0	0	0	0	0	0	0	0	0	0	1	0	0	0	1	0	0	0	0	0	0	0	0	0	0	0	0	0	12
TOP_JPL ADASYN_10	0	0	0	0	0	0	0	0	0	0	0	0	0	0	0	0	0	0	1	1	0	1	1	1	0	1	0	1	1	0	0	0	0	0	0	0	0	0	0	0	0	0	0	0	0	0	0	0	0	0	1	1	1	1	0	0	0	12
RIGHT_GFZ ADASYN_4	0	0	0	0	0	1	1	1	1	1	0	1	1	0	0	1	0	0	0	0	0	0	1	0	0	0	0	0	0	0	0	0	0	0	0	0	0	1	0	0	0	0	0	0	0	0	0	0	0	0	0	0	0	0	0	0	0	10
Bottom_GFZ SMOTE_10	1	1	0	0	0	1	0	0	1	1	1	0	0	1	0	0	1	0	0	0	0	0	0	0	1	0	0	0	0	0	0	0	0	0	0	0	0	0	0	0	0	0	0	0	0	0	0	0	0	0	0	0	0	0	0	0	0	9
Surrounding_UTCSR 60 SMOTE_10_120min	0	0	0	0	1	1	0	0	1	1	0	0	0	0	0	0	1	0	0	0	0	0	0	0	1	0	1	0	0	0	0	0	0	0	0	0	0	0	0	1	0	0	0	1	0	0	0	0	0	0	0	0	0	0	0	0	0	9
Surrounding_UTCSR 96 ADASYN_10_60min	0	0	0	0	0	0	0	0	0	0	0	1	1	0	0	0	0	0	0	0	1	0	0	0	0	0	0	0	0	1	0	0	0	0	1	0	0	1	1	0	0	0	0	0	0	0	0	0	0	0	0	0	0	0	0	1	1	9
Bottom_GFZ ADASYN_10	1	0	0	0	0	1	0	0	1	1	1	0	0	1	0	0	1	0	0	0	0	0	0	0	1	0	0	0	0	0	0	0	0	0	0	0	0	0	0	0	0	0	0	0	0	0	0	0	0	0	0	0	0	0	0	0	0	8
Bottom_GFZ SMOTE_4	1	0	0	0	0	1	0	0	1	1	1	0	0	1	0	0	1	0	0	0	0	0	0	0	0	0	0	0	0	0	0	0	0	0	0	0	0	0	0	0	0	0	0	0	0	0	0	0	0	0	0	0	0	0	0	0	0	7
Bottom_JPL ADASYN_10	1	1	0	0	0	1	0	0	1	0	1	0	0	1	0	0	0	0	0	0	0	0	0	0	0	0	0	0	0	0	0	0	0	0	0	0	0	0	0	0	0	0	0	0	0	0	1	0	0	0	0	0	0	0	0	0	0	7
Total number of times the field detected	5	4	2	4	6	11	5	4	18	16	5	3	3	6	1	3	5	2	3	6	3	7	7	4	8	5	4	8	7	6	7	3	7	9	8	7	5	8	6	10	4	4	3	9	5	6	1	7	7	6	2	6	10	8	1	5	9	

### Experimentation with Different Test Dataset

**Table 7:** Matrix with the minimum number of best-performing models detecting the maximum number of deposits in the testing region for the new test dataset from Harvard.

Models	Petroleum Field No																																																									
	1	2	3	4	5	6	7	8	9	10	11	12	13	14	15	16	17	18	19	20	21	22	23	24	25	26	27	28	29	30	31	32	33	34	35	36	37	38	39	40	41	42	43	44	45	46	47	48	49	50	51	52	53	54	55	56	57	Count
RIGHT_GFZ ADASYN_10	0	0	0	1	1	1	1	1	1	1	0	0	0	0	0	0	0	0	0	0	0	1	1	1	0	0	0	1	0	0	0	0	1	1	0	1	1	1	1	1	0	0	0	1	0	0	0	0	1	1	0	1	1	1	0	0	1	25
RIGHT_GFZ SMOTE_10_150min	0	0	0	1	1	0	0	0	1	1	0	0	0	0	0	0	0	0	0	0	0	0	0	0	0	0	0	0	1	1	1	0	1	1	1	1	0	0	0	1	1	1	1	1	1	1	0	1	0	0	0	0	1	1	0	1	1	23
RIGHT_UTCSR 60 SMOTE_10	1	1	0	0	0	0	0	0	1	1	0	0	0	0	0	1	0	1	1	1	1	1	0	0	1	1	0	1	1	1	1	0	1	0	1	1	0	1	0	1	0	0	0	0	0	1	0	0	1	0	0	0	0	0	0	0	0	23
RIGHT_JPL ADASYN_10	0	0	1	0	0	0	0	0	1	1	0	0	0	0	0	0	0	0	0	1	0	1	1	0	0	1	0	1	1	1	0	0	1	0	1	0	0	1	1	0	0	0	0	0	0	1	0	1	0	0	0	0	1	1	1	1	1	21
TOP_JPL SMOTE_10	0	0	0	0	0	0	0	0	0	0	0	0	0	0	0	0	0	0	0	1	0	1	1	1	0	1	1	1	1	0	1	1	1	1	1	0	0	0	0	0	0	0	0	0	0	0	0	0	0	0	1	1	1	1	0	0	0	17
Surrounding_UTCSR 96 SMOTE_10_1000min	0	0	0	0	0	0	1	1	0	0	1	1	1	1	0	0	0	0	0	0	0	0	0	0	0	0	0	0	0	1	1	0	0	1	1	0	0	0	0	0	0	0	0	1	1	0	0	1	0	0	0	0	0	0	0	1	1	15
RIGHT_UTCSR 60 ADASYN_10	0	1	0	0	0	0	0	0	1	0	0	0	0	1	1	0	1	1	1	0	1	0	0	0	1	0	1	0	0	0	0	0	0	0	0	0	0	0	0	1	0	0	0	1	0	0	0	0	0	0	0	0	0	0	0	0	0	12
Bottom_JPL ADASYN_10	1	1	0	0	0	1	0	0	1	0	1	0	0	1	0	0	0	0	0	0	0	0	0	0	0	0	0	0	0	0	0	0	0	0	0	0	0	0	0	0	0	0	0	0	0	0	1	0	0	0	0	0	0	0	0	0	0	7
Total	2	3	1	2	2	2	2	2	6	4	2	1	1	3	1	1	1	2	2	3	2	4	3	2	2	3	2	4	4	4	4	1	5	4	5	3	1	3	2	4	1	1	1	4	2	3	1	3	2	1	1	2	4	4	1	3	4

**Table 8:** Comparison between our proposed method with other existing related research studies.

Method	Regions Under Study	Covered Area ($km^2$)	Grid Size	Number of Points	Data Source(s)	Basis of Prediction	Basis of Mathematical Expansion	Finding(s)
Zeng et al. 2002^8^	Shengli oil field, East China	800	0.5km $\times$ 0.25km	Not Mentioned	Shengli Petroleum Administration Bureau, China - local	Normalized Full Gradient	Fourier Series	Identification of the center of existing petroleum deposits
	Dabeil Area, East China	30	0.09km $\times$ 0.09km	3275				
Aghajani et al. 2011^7^	Tabas Basin in Yazd province, Eastern Iran	4,545	1.5km $\times$ 3km	1,115	Geophysics department of National Iranian Oil Company (NIOC) - local	Normalized Full Gradient	Fourier Series	Identification of the center of an existing petroleum deposit
Perry et al. 2011^31^	Kurdistan, Northern Iraq	32000	N/A	N/A	Landsat Enhanced Thematic Mapper, Advanced Spaceborne Thermal Emission and Reflection Radiometer	Landsat visible and near-IR (VNIR) bands 1, 2, and 3	ENVI Image Processing Software	Evidence of Hydrocarbon Seepage
Behadili et al. 2019^30^	AL Nasiriya, Southern Iraq	19200	N/A	N/A	Landsat-7 Enhanced Thematic Mapper	Stefan - Boltzmann law	Environment for Visualizing Images (ENVI 5.3)	Uncovers, and estimates several unexplored oil and gas fields
Our proposed method	Iraq and its surrounding regions	14,889,269 as training, 851,131 as testing	0.1$^\circ$ x 0.1$^\circ$, or ((6.7 - 10.7km) $\times$ 11km)	146,772 as training, 8,415 as testing	UTCSR, JPL, and GFZ - global	Gravity Gradient Tensor	Legendre Polynomial	Prediction of new prospective petroleum deposits

**Figure 12:**
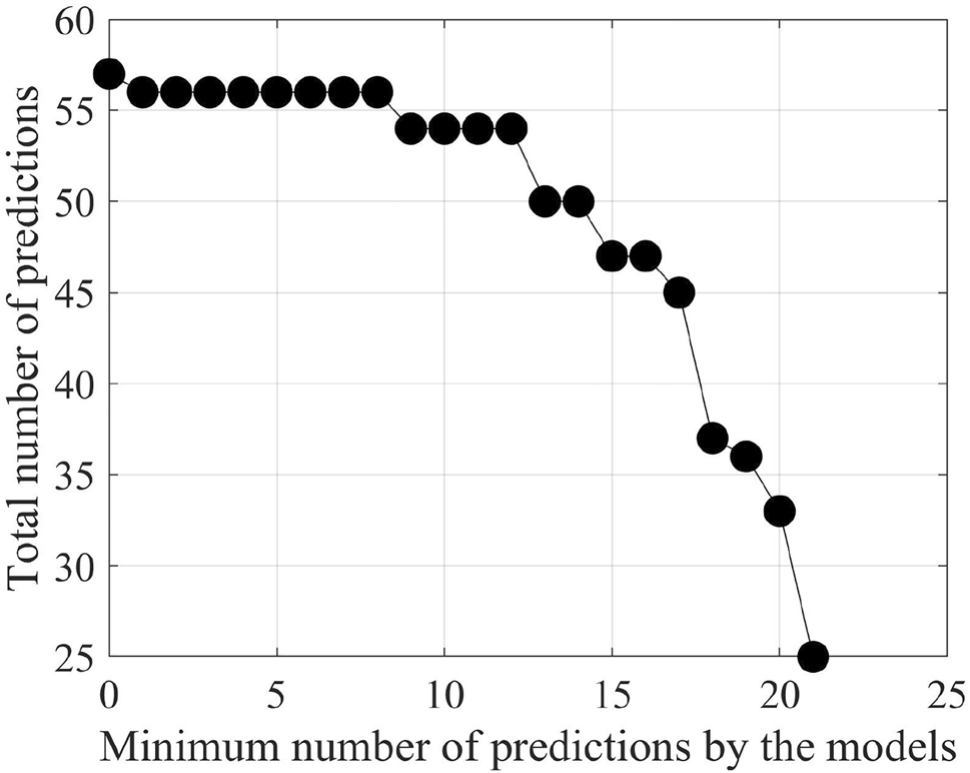
The process of finding the threshold for the new test dataset from Harvard.

**Figure 13:**
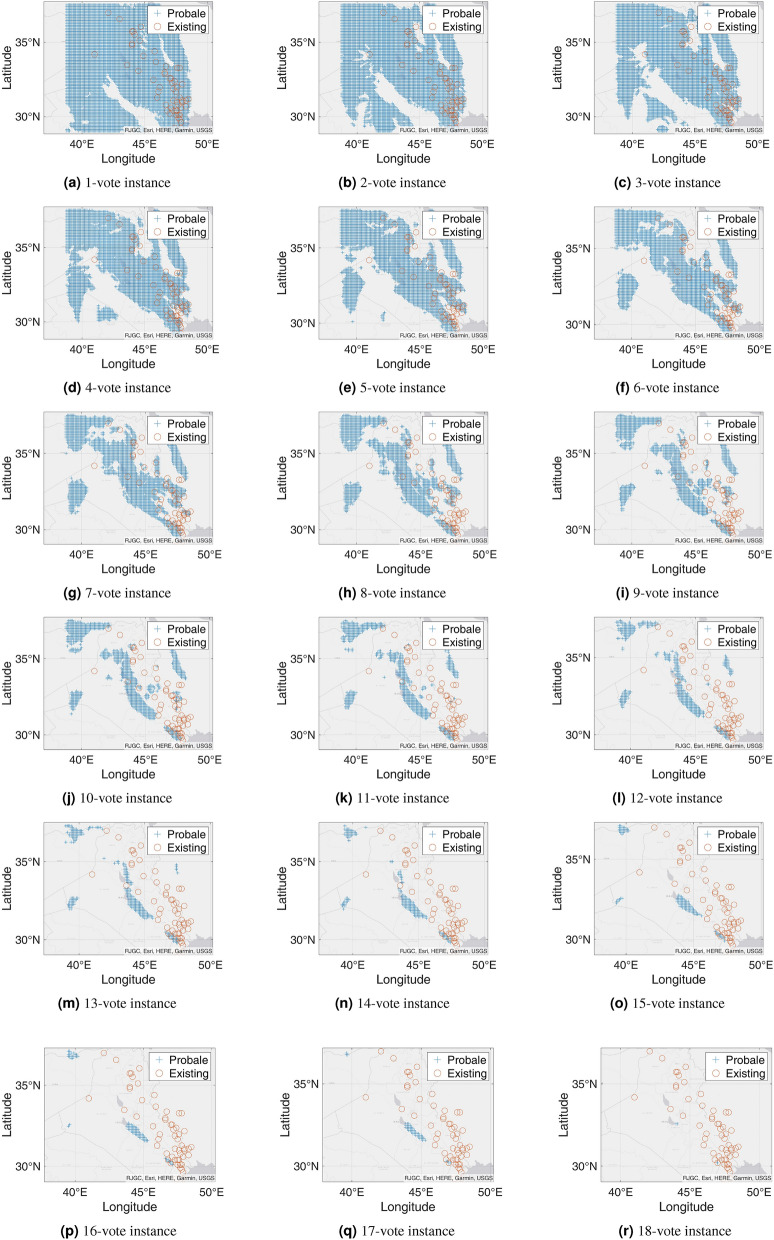
Probable petroleum deposits based on the existence of 1 to 18 votes over our 22 best-performing models from the matrix-based analysis over the new test dataset from Harvard (the figures are created using MATLAB 2021a^51^).

**Figure 14:**
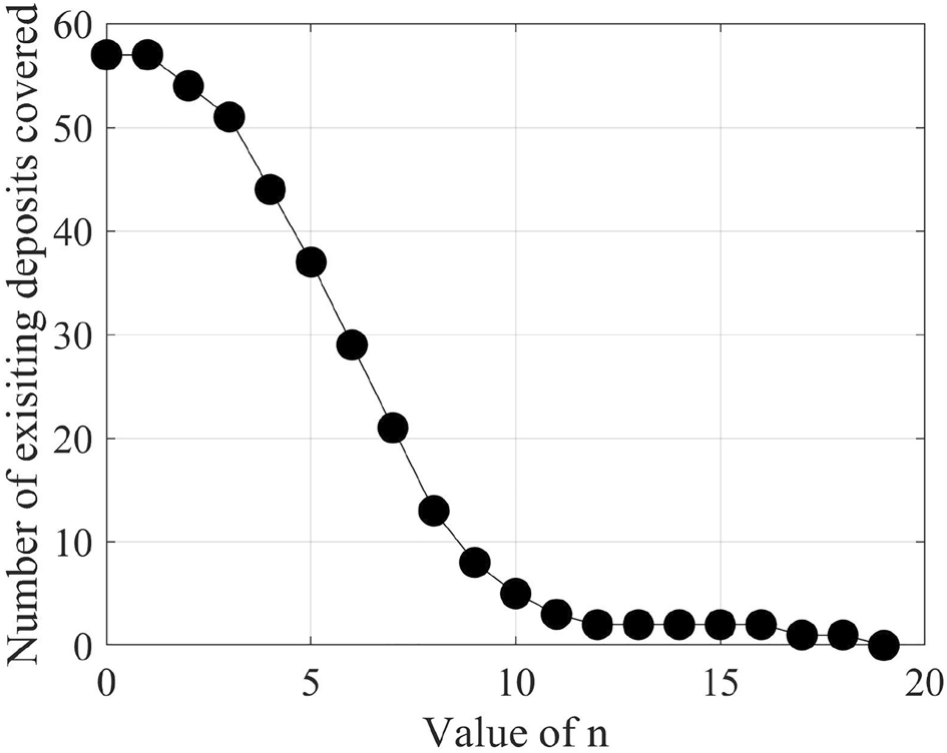
Change in the number of existing petroleum deposits covered by n-vote instances with an increasing value of n for the new test dataset from Harvard.

**Figure 15:**
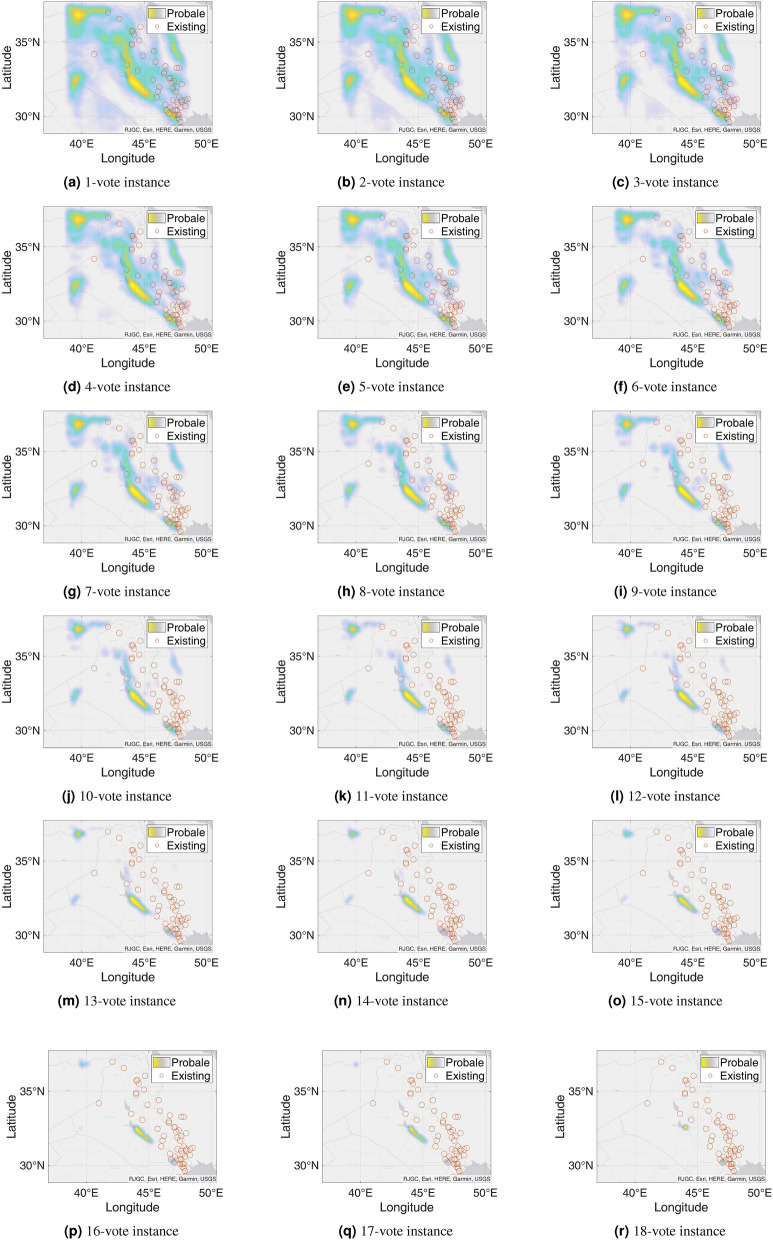
Probable petroleum deposits based on the frequency of 1 to 18 votes over our 22 best-performing models from the matrix-based analysis over the new test dataset from Harvard (the figures are created using MATLAB 2021a^51^).

**Figure 16:**
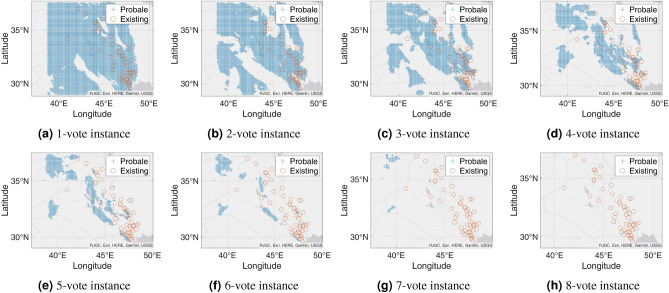
Probable petroleum deposits based on the existence of 1 to 8 votes over our eight best-performing models found from the graph-based analysis for the new testing dataset from Harvard (the figures are created using MATLAB 2021a^51^).

**Figure 17:**
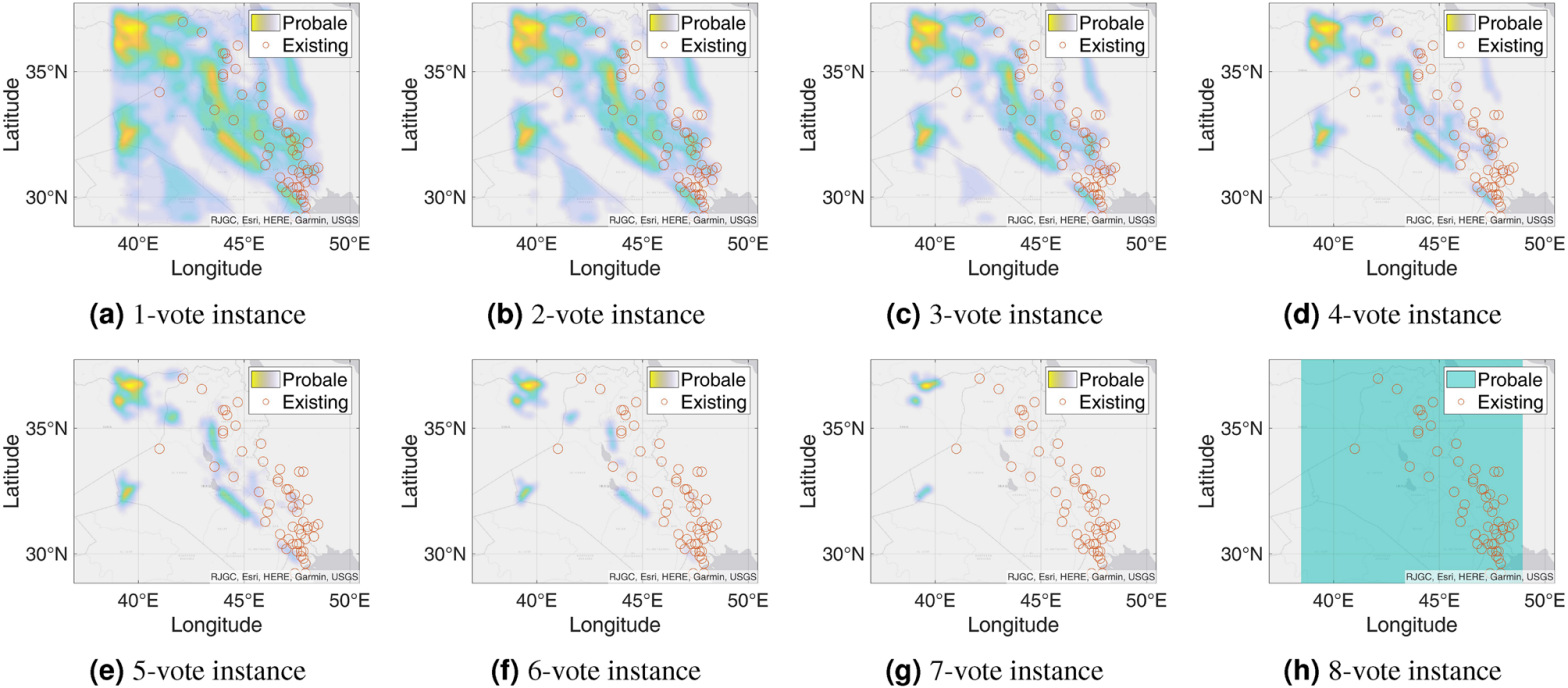
Probable petroleum deposits based on the frequency of 1 to 8 votes over our eight best-performing models found from the graph-based analysis for the new testing dataset from Harvard (the figures are created using MATLAB 2021a^51^).

In our experimentation presented above, we use a test dataset for existing petroleum deposits from PRIO^9^. Next, to test the generalizability of our proposed method, we perform our experimentation over a different test dataset. We collect the new test dataset from Harvard ArcGIS WorldMap^10^. Here, the locations of the existing petroleum deposits matched with a similar dataset we find in the experiment done by Ameri et al.^53^. In the case of the new test dataset from Harvard, the testing region is the same as our earlier experiment. Here, only the existing petroleum deposits have got changed from what we find in the dataset from PRIO.

We carefully compare these two test datasets. We find that the test dataset from PRIO gives us 26 existing petroleum deposits, whereas, the Harvard test dataset gives us 57 existing petroleum deposits in the same region. A potential reason behind this happening is the fact that, PRIO generally considers adjacent deposits as a single point, whereas, Harvard considered them as separate ones. Accordingly, over the existing petroleum deposits found in the dataset from Harvard, we can find many petroleum deposits adjacent to each other.

We apply all the best-performing models to our newly-considered test dataset from Harvard. This time, some of the best-performing models can predict at most 25 of existing petroleum deposits. In addition, here, each of the 57 existing petroleum deposits is detected by at least one of the best-performing models. Again, this time, the predicted petroleum deposits vary for different models mimicking our earlier case.

We prepare a matrix with the outputs of all the 80 best-performing models for the test dataset from Harvard. This time, we find that the threshold value is seven for detecting all the existing petroleum deposits in the testing field. Therefore, we keep those models that can detect at least seven existing petroleum deposits from our new test dataset. Figure 12 shows how the threshold value gets selected through the process of getting the maximum number of predicted petroleum deposits saturated.

#### n-Voting over Results of ML Methods to the New Testing Dataset

We get a total of 25 best-performing models that can detect seven or more existing petroleum deposits from the new test dataset from Harvard. We apply the n-voting mechanism to these 25 best-performing models. We find that, among the 25 best-performing models, 22 are enough to detect all the existing petroleum deposits. Each of the remaining three models detects seven existing petroleum deposits. Therefore, we remove them from our solution list and apply n-Voting to the remaining 22 models.

Table 6 shows the matrix with the best-performing 22 models that can detect at least seven existing petroleum deposits from the new testing dataset from Harvard individually. The 22 models, in combination, can detect all of the petroleum deposits using our proposed method. Over these models, we apply 1-voting, 2-voting, 3-voting, etc., up to 22-voting. We find that, up to 18-voting, we get prospective results. However, in the cases of 19-, 20-, 21-, and 22-voting, we get no coverage over the existing deposits. Hence, from 19 votes, not a single existing field can be detected by all the methods. This is justified, because, from the last row of Table 6, we can see that an existing field is detected a maximum of 18 times by all the models. Therefore, it is enough to apply up to 18-voting to cover the deposits. Figure 13 shows the outcomes of the n-voting mechanism to our new test dataset. The best case in Fig. 13 (i.e., Fig. 13a or the 1-vote instance) covers 57 out of the existing 57 deposits. In the case of 2-vote instance (i.e., Fig. 13b), the coverage decreases to 54 out of the existing 57 deposits, and so on. Figure 14 presents the scenario. On the other hand, Fig. 15 shows the heatmaps of the n-Voting mechanism.

#### Graph-based Analysis for Maximum Coverage with Minimum Number of Resulting Model Outputs for the New Test Dataset

Similar to our earlier experiment, we perform the graph-based analysis using Algorithm 1. This time, we get a total of eight best-performing models to cover all the existing petroleum deposits in our new testing dataset. Table 7 shows the matrix of detecting the existing petroleum deposits by these eight models. Again, we perform n-voting over these eight best-performing models. Here, we find similar results as already obtained in the earlier case. Figure 16 shows the results we find from n-voting over these eight models as well as the graph-based analysis. Additionally, Fig. 17 shows the heatmaps of the n-voting mechanism when applied to these eight models found from the graph-based analysis.

## Discussion

We perform our study using publicly available satellite data over the region of Iraq. As this data is available for the whole earth, therefore, we can extend the study area anytime. In this regard, a further implication of our study is discussed next in this section. And the comparison with other studies is presented.

### Further Implications of Our Study

We have used GGT anomaly as the main fuel of our working technique for the purpose of our study. We combined the GGT value with the location of the existing petroleum deposits in our area of focus, and thus, created new labeled datasets. We use Auto-WEKA for the shake of running the 28 standard ML methods. Auto-WEKA is one of the leading tools for modeling and testing ML methods. The followings are some different alterations that could be possible in the processes of our study:

#### Use of GGT Instead of Gravity Information Due to Sensitivity

We took spherical harmonic from the GRACE satellite and calculate the GGT from that data. Gravity information can also be calculated from the GRACE satellite data. However, since GGT is the 2nd derivative of gravitational force, therefore it is a more sensitive property than the gravity property of the earth and can sense a very small anomaly in gravitational field^39^.

#### Change of Study Region

In this study, we focus on the region of Iraq and its surrounding areas. Note that, we took the region because there are a number of existing petroleum deposits in our study area. This helped our method to learn the property of the earth at the location of existing petroleum deposits and predict a new one using the learning. Since our method is a generalized one, therefore, anyone can use this method anywhere in the world. In that case, he needs to calculate the GGT for that new region. Then, he also needs to combine the GGT with the existing petroleum deposits of that region and label them. Moreover, he also needs to oversample the training data he would make. If the existing petroleum deposits in that area are much less than in Iraq and its surroundings, then the oversampling percentage will get high, which will create the chance of more false predictions. We would like to test our method for some other areas of the earth as a future work of this study.

### Comparison with Other Existing Related Studies

We compare our proposed method with other existing recent related studies. Table 8 presents an overview of the comparison. Here, we perform the comparison based on the region under study, covered area, grid size, the number of data points under consideration, data source(s), the basis of prediction, the basis of mathematical expansion, and finding(s).

As shown in the table, both the studies by Zeng et al.^8^ and Aghajani et al.^7^ explored gravity data using Normalized Full Gradient (NFG) of gravity to locate wells in already-known oil reservoirs. Besides, Perry et al.^31^ studied satellite images from the United States Geological Survey (USGS) to locate the evidence of hydrocarbon seepage. They used Landsat images with the enhanced thematic mapper and advanced spaceborne thermal emission. Their basis of the prediction is Landsat Visible and Near-IR (VNIR) Band 1, 2, and 3. Similarly, Behadili et al.^30^ studied Landsat-7 images from USGS to locate the unexplored oil and gas fields in the AL Nasiriya city of Southern Iraq. Both of these studies used ENVI as image processing software to study satellite images.

On the other hand, in our proposed method, we use Gravity Gradient Tensor as a new basis of prediction and try to find a new petroleum reservoir. Besides, the first two studies^7,8^ used locally collected gravity data in their prediction tasks. On the contrary, we use gravity data collected by the GRACE satellite, which is processed by three different highly-reputed organizations. Besides, gravity data is universally available for the whole earth, as they are collected through the satellite. Due to the widespread availability of satellite data, we can apply our proposed method anywhere in the world. Nonetheless, all the mentioned existing studies covered very small areas in their explorations, whereas, our study covers a large area for its exploration. With all these comparisons, we find our study presents a more comprehensive approach, which will advance the knowledge in literature and open the door to a pervasive mechanism for predicting new underground petroleum deposits.

## Future Work

We have different types of plans for our future work. In this study, we have used GRACE satellite data as the primary data source. We plan to collect data from other satellites or GIS, i.e., GOCE (Gravity field and steady-state Ocean Circulation Explorer), Landsat-8, etc., and combine them with GRACE satellite data. Besides, in this study, we focus on the region of Iraq and its surroundings. We plan to study other regions in the future. Moreover, we plan to include some different environmental aspects in this study, i.e., the earth’s magnetic field.

## Conclusion

Existing exploratory techniques of detecting petroleum deposits demand a long process and incur a high budget. Many potential areas can not be investigated due to these constraints. As a remedy to this situation, we propose a new method of predicting the location of a petroleum deposit based on publicly available data sensed by an open satellite named Gravity Recovery and Climate Experiment (GRACE). Leveraging the GRACE data, we propose to calculate the gravity gradient tensor of the earth over the region under focus. To demonstrate the efficacy of our proposed method, we choose Iraq as an experimental area considering the existence of a good number of petroleum deposits within it. Here, through incremental improvement over our proposed methodologies (combining machine learning, graph-based analysis, and the newly-proposed OR-nAND method altogether), we can predict 25 out of 26 existing petroleum deposits reported by a dataset from PRIO within the area under our study. We demonstrate the generalizability of our proposed methodologies through exploring another dataset from Harvard ArcGIS WorldMap resulting in similar outcomes as already obtained for the dataset from PRIO.

It is worth mentioning that our proposed method does not replace the existing technology. Rather, our method can narrow down and spot out the search area with a higher chance of success. Therefore, if we can narrow our search area with a greater chance of success, then more potential places can be explored with seismic technology and other advanced technologies reducing the budget required for the purpose of searching while uplifting the chance of getting petroleum deposits with less number of physical explorations.

## Supplementary Information

Supplementary Information 1.

Supplementary Information 2.

## Data Availability

All data generated or analyzed during this study are included in this published article (and its Supplementary Information files).
