# Supplementary material for: A ubiquitous method for predicting underground petroleum deposits based on satellite data
Source: Sci Rep. 2023 Apr 24;13:6638. doi: 10.1038/s41598-023-32054-0 (PMC10125970; doi:10.1038/s41598-023-32054-0)
Supplement: Supplementary file 2 — Supplementary Information 2. [file 41598_2023_32054_MOESM2_ESM.pdf]

Supplementary Equations

Derivation of Gravitational Gradient Tensor from Spherical Harmonic Coefficients

The gravitational field of any point on the earth can be computed as follows.

$$V = \frac{GM}{r} \times \left(\sum_{n=2}^{\infty} \sum_{m=0}^{n} \left(\frac{a_e}{r} \right)^n \times \left(C_{nm} \times \cos(m\lambda) + S_{nm} \times \sin(m\lambda) \right) \times P_{nm} \times \cos\theta \right) \tag{1}$$

Here, C_{nm} and S_{nm} are the spherical harmonic (SH) coefficients[1], which describe the mass distribution within the earth. a_e is the equatorial radius. r, θ, and λ are the radius, colatitude, and longitude respectively. P_{nm} is the associated Legendre function. GM is the gravitational constant multiplied by the mass of the earth.

In Eq. 1, equatorial radius a_e eventually gets replaced by R, as the sphere is the earth in this case. Accordingly, based on Eq. 1, the first derivatives of V with respect to r, θ, and λ can be derived as follows.

$$V_r(r,\theta,\lambda) = -\frac{GM}{R^2} \left(\sum_{n=2}^{\infty} (n+1) \left(\frac{R}{r} \right)^{n+2} \times \sum_{m=0}^{n} \left(C_{nm} \times \cos(m\lambda) + S_{nm} \times \sin(m\lambda) \right) \times P_{nm} \times (\cos\theta) \right) \tag{2}$$

$$V_\theta(r,\theta,\lambda) = -\frac{GM}{R} \left(\sum_{n=2}^{\infty} \left(\frac{R}{r} \right)^{n+1} \times \sum_{m=0}^{n} \left(C_{nm} \times \cos(m\lambda) + S_{nm} \times \sin(m\lambda) \right) \times P'_{nm}(\cos\theta) \times \sin\theta \right) \tag{3}$$

$$V_\lambda(r,\theta,\lambda) = \frac{GM}{R} \left(\sum_{n=2}^{\infty} \left(\frac{R}{r} \right)^{n+1} \times \sum_{m=0}^{n} m\left(-C_{nm} \times \sin(m\lambda) + S_{nm} \times \cos(m\lambda) \right) \times P_{nm} \times (\cos\theta) \right) \tag{4}$$

Accordingly, the second derivatives of V with respect to r, θ, and λ are as follows.

$$V_{rr}(r,\theta,\lambda) = \frac{GM}{R^3} \left(\sum_{n=2}^{\infty} (n+1)(n+2) \left(\frac{R}{r} \right)^{n+3} \times \sum_{m=0}^{n} \left(C_{nm} \times \cos(m\lambda) + S_{nm} \times \sin(m\lambda) \right) \times P_{nm} \times (\cos\theta) \right) \tag{5}$$

$$V_{r\theta}(r,\theta,\lambda) = \frac{GM}{R^2} \left(\sum_{n=2}^{\infty} (n+1) \left(\frac{R}{r} \right)^{n+2} \times \sum_{m=0}^{n} \left(C_{nm} \times \cos(m\lambda) + S_{nm} \times \sin(m\lambda) \right) \times P'_{nm} \times (\cos\theta) \sin\theta \right) \tag{6}$$

$$V_{r\lambda}(r,\theta,\lambda) = \frac{GM}{R^2} \left(\sum_{n=2}^{\infty} (n+1) \left(\frac{R}{r} \right)^{n+2} \times \sum_{m=0}^{n} m\left(C_{nm} \times \sin(m\lambda) - S_{nm} \times \cos(m\lambda) \right) \times P_{nm} \times (\cos\theta) \right) \times \tag{7}$$

$$V_{\theta\theta}(r,\theta,\lambda) = \frac{GM}{R} \left(\sum_{n=2}^{\infty} \left(\frac{R}{r} \right)^{n+1} \times \sum_{m=0}^{n} \left(C_{nm} \times \cos(m\lambda) + S_{nm} \times \sin(m\lambda) \right) \times \left(P''_{nm} \times \cos\theta \times \sin^2\theta - P'_{nm} \times \cos^2\theta \right) \right) \tag{8}$$

$$V_{\theta\lambda}(r,\theta,\lambda) = \frac{GM}{R} \left(\sum_{n=2}^{\infty} \left(\frac{R}{r} \right)^{n+1} \times \sum_{m=0}^{n} m \times \left(C_{nm} \times \sin(m\lambda) - S_{nm} \times \cos(m\lambda) \right) \times P'_{nm} \times \cos\theta \times \sin\theta \right) \tag{9}$$

$$V_{\lambda\lambda}(r,\theta,\lambda) = -\frac{GM}{R} \left(\sum_{n=2}^{\infty} \left(\frac{R}{r} \right)^{n+1} \times \sum_{m=0}^{n} m^2 \times \left(C_{nm} \times \cos(m\lambda) + S_{nm} \times \sin(m\lambda) \right) \times P_{nm} \times \cos\theta \right) \tag{10}$$

The gravitational gradients (full tensor) in the local North–East–Down (NED)[1] frame can be further derived as follows.

$$V_{xx}(r,\theta,\lambda) = \frac{1}{r}V_r(r,\theta,\lambda) + \frac{1}{r^2}V_{\theta\theta}(r,\theta,\lambda) \tag{11}$$

$$V_{xy}(r,\theta,\lambda) = V_{yx}(r,\theta,\lambda) = \frac{1}{r^2 \sin\theta}(-\cot\theta V_\lambda(r,\theta,\lambda) + V_{\theta\lambda}(r,\theta,\lambda)) \tag{12}$$

$$V_{xz}(r,\theta,\lambda) = V_{zx}(r,\theta,\lambda) = \frac{1}{r}V_{r\theta}(r,\theta,\lambda) - \frac{1}{r^2}V_\theta(r,\theta,\lambda) \tag{13}$$

$$V_{yy}(r,\theta,\lambda) = \frac{1}{r}V_r(r,\theta,\lambda) + \frac{1}{r^2}\cot\theta V_\theta(r,\theta,\lambda) + \frac{1}{r^2\sin^2\theta}V_{\lambda\lambda}(r,\theta,\lambda) \tag{14}$$

$$V_{yz}(r,\theta,\lambda) = V_{zy}(r,\theta,\lambda) = \frac{1}{r\sin\theta}\left(V_{r\lambda}(r,\theta,\lambda) - \frac{1}{r}V_\lambda(r,\theta,\lambda)\right) \tag{15}$$

$$V_{zz}(r,\theta,\lambda) = V_{rr}(r,\theta,\lambda) \tag{16}$$

References

1. Wang, L., Shum, C. K. & Jekeli, C. Gravitational gradient changes following the 2004 december 26 sumatra–andaman earthquake inferred from grace. *Geophys. J. Int.* **191**, 1109–1118 (2012). URL http://dx.doi.org/10.1111/j.1365-246X.2012.05674.x. DOI 10.1111/j.1365-246X.2012.05674.x.
